# Landscape of Transposable Elements Focusing on the B Chromosome of the Cichlid Fish *Astatotilapia latifasciata*

**DOI:** 10.3390/genes9060269

**Published:** 2018-05-23

**Authors:** Rafael L. B. Coan, Cesar Martins

**Affiliations:** Department of Morphology, Institute of Biosciences, São Paulo State University (UNESP), 18618-689 Botucatu, SP, Brazil; rafaelbcoan@gmail.com

**Keywords:** repetitive elements, RNA-Seq, genomics, evolution, cytogenetics, supernumerary elements, extra chromosomes

## Abstract

B chromosomes (Bs) are supernumerary elements found in many taxonomic groups. Most B chromosomes are rich in heterochromatin and composed of abundant repetitive sequences, especially transposable elements (TEs). B origin is generally linked to the A-chromosome complement (A). The first report of a B chromosome in African cichlids was in *Astatotilapia latifasciata*, which can harbor 0, 1, or 2 Bs Classical cytogenetic studies found high a TE content on this B chromosome. In this study, we aimed to understand TE composition and expression in the *A. latifasciata* genome and its relation to the B chromosome. We used bioinformatics analysis to explore the genomic organization of TEs and their composition on the B chromosome. The bioinformatics findings were validated by fluorescent in situ hybridization (FISH) and real-time PCR (qPCR). *A. latifasciata* has a TE content similar to that of other cichlid fishes and several expanded elements on its B chromosome. With RNA sequencing data (RNA-seq), we showed that all major TE classes are transcribed in the brain, muscle, and male and female gonads. An evaluation of TE transcription levels between B- and B+ individuals showed that few elements are differentially expressed between these groups and that the expanded B elements are not highly transcribed. Putative silencing mechanisms may act on the B chromosome of *A. latifasciata* to prevent the adverse consequences of repeat transcription and mobilization in the genome.

## 1. Introduction

B chromosomes (Bs) are supernumerary elements in addition to autosomal (A) chromosomes and have been observed in several species of animals, plants, and fungi. B chromosomes have an evolutionary pathway distinct from that of A chromosomes and a non-Mendelian form of inheritance [1]. Diverse publications have shown that Bs can have neutral, deleterious, or beneficial effects on their hosts: the presence of an additional B chromosome is correlated to sex determination in the cichlid fish *Lithochromis rubripinnis*, V-shaped phenotype in the common frog *Rana temporaria* and antibiotic resistance in the fungus *Nectria haematococca* [1,2,3,4].

B chromosomes are usually heterochromatic due to the abundance of repetitive elements in their composition [5]. Regardless of the predominance of repetitive content on Bs, they can carry genes with different levels of integrity, including fully transcribed copies. Gene copies found on B chromosomes can modulate the gene expression of A-complement genes and even influence metabolic pathways [6,7,8]. B chromosomes usually consist of a mosaic of sequences from the A complement; B origin is generally linked to genomic instability in the A chromosomes and involves the formation of a proto-B and later expansion of repetitive elements [9,10,11]. Repetitive elements, including transposable elements (TEs—DNA transposons and retrotransposons) [12,13,14,15], represent a large portion of most eukaryotic genomes and are also a major component of B chromosome constitution [3,13]. Furthermore, gene clusters such as U2 small nuclear RNA (snRNA) [16], 18S ribosomal RNA (rRNA), and H1 [11], H3, and H4 histones [17] have also been found on Bs in many species. Classical cytogenetic analysis is used to locate B chromosome origins on different A-complement pairs with the use of probes from multigenic families and TEs [11,18,19,20,21]. The TE content on B chromosomes is related to the TE content of the A complement, and repeat expansion on Bs is a common characteristic of these supernumerary elements, caused by lack of recombination and low selective pressure [3]. For example, on the B chromosome of rye, accumulation of satellite DNA, Ty1/Copia, a long terminal repeat (LTR) retrotransposon, and a few unclassified sequences are present [22]. On the B chromosome of the fish *Alburnus alburnus*, an expanded Ty3/Gypsy sequence showed similarity to the reverse transcriptase coding gene [23].

In addition to the accumulation of repetitive elements on Bs, evidence also exists that those elements can be transcribed. The StarkB element specific to the maize B chromosome has variable expression among individuals and loci [24]. In a different study, this element was found to be expressed with the Gypsy and Copia TEs. In fact, StarkB expression showed evidence of dose dependence; its expression increased as the number of Bs increased. This result demonstrates the influence of the number of B chromosomes in the transcription of their sequences [25]. Another recent study found differentially expressed repetitive sequences between 0B and 4B individuals in rye. The same study also noted differences in transcription among the different tissues [26].

Among African cichlids, B chromosomes have been observed in twenty species [2,19,27,28,29], being first described in *Astatotilapia latifasciata*, which may have 0, 1, or 2 Bs [27]. Cichlid fish from the Great Lakes of East Africa have experienced a rapid adaptive radiation and the B chromosome occurrence represents a new enigma to be investigated in the focus of evolutionary biology [30,31,32]. The Bs of *A. latifasciata* are among the largest B chromosomes investigated to date. They are heterochromatic and contain abundant repetitive elements, as evidenced by classical cytogenetic mappings of 18S ribosomal DNA (rDNA) and the Rex1 and Rex3 elements [19,27]. A recent next-generation sequencing (NGS) analysis also revealed an accumulation of diverse classes of repetitive sequences on the B chromosome of this species [10]. Thus, our study aims to understand TE content, distribution, and transcription on the *A. latifasciata* genome and its impact on the B chromosome. We used a combination of classical molecular cytogenetics and NGS to evaluate the TE landscape of the species and find representative sequences on the B chromosome. We analyzed the transcription levels of the repeats and their possible relations to B-enriched sequences. Understanding the repeat content of the A complement and its relation to the B chromosome will help elucidate the constitution and perpetuation of the B chromosome in *A. latifasciata*, as well as its influence on the cell biology of the species.

## 2. Materials and Methods

### 2.1. Samples

All animal samples were obtained from the fish facility of the Integrative Genomics Laboratory, São Paulo State University, Botucatu, Brazil. We complied with the ethical principles adopted by the Brazilian College of Animal Experimentation, with approval from the Institute of Biosciences/UNESP São Paulo State University ethics committee (protocol no 486-2013). We used samples from males and females with 1 and 2 B chromosomes (B+) or without Bs (B-). Samples were genotyped as B- and B+ by PCR with specific primers for B chromosome presence or absence [33].

All datasets for bioinformatics analysis were previously sequenced. DNA sequencing was performed in four male (M1-0B, M2-1B, M3-1, and M4-2B) and two female samples (F1-0B and F2-1B) from two previous studies [10,34]. The sequencing data included 0B, 1B, and 2B samples. RNA sequencing (RNA-seq) was performed for three tissues: brain, muscle, and gonads. Within each tissue, six samples were 1B and six 0B. Each B condition (1B or 0B) had three males and three females, with 36 total RNA-seq samples. All data are available in SaciBase (sacibase.ibb.unesp.br/). For fluorescent in situ hybridization (FISH), we used a 1B sample confirmed though molecular PCR and cytogenetic analysis. For real-time PCR (qPCR), we used B- (sample ID 04) and B+ (sample IDs 907, 908, 910, 913, 918, and 919) samples confirmed by PCR.

With the exception of M4-2B, a B+ samples with 2B chromosomes, all genomic B+ samples in this study were 1B. We prioritize the B- (0B) and B+ (1B or 2B) nomenclature though the text, but when necessary we will indicate the number of B chromosomes under analysis and discussion (0B, 1B, or 2B). All B+ samples used for expression analysis were 1B. All investigated samples are summarized in Supplementary Table S1.

### 2.2. Pipelines for Repeat Identification and Repeat Landscape Construction

To estimate the repetitive content in the genome of *A. latifasciata*, we used an assembled B- genome containing male and female reads as reference [34]. We first created a custom repeat library with RepeatModeler 1.0.8 [35] according to the instructions and using the default parameters. Any ID issue in the created *fasta* file was manually checked. We merged the custom repeat library with Repbase Update 20150807 [36] in order to obtain a comprehensive repeat library for input to RepeatMasker. Despite the manual curation, some sequences maintained the “Unknown” status. In the second step of the repeat identification, the merged library was used as input for RepeatMasker 4.0.5 [37] to search for repeat copy number and organization in the assembled genome. RepeatMasker was run with the “slow (-s)”, “align (-a)”, and “library (-lib)” parameters. To summarize the RepeatMasker results, we used “buildSummary.pl”, a Perl script from the RepeatMasker package. The output files from RepeatMasker were also used as input for the “createrepeatlandscape.pl” and “calcdivergencefromalign.pl” scripts to calculate the Kimura divergence values and plot the repeat landscape. Both are helper Perl scripts from the RepeatMasker package.

### 2.3. Comparative Analysis Pipeline

For the first search of expanded elements on the B chromosome of *A. latifasciata*, we used RepeatExplorer [38], which performs a graph-based clustering of raw Illumina reads. RepeatExplorer uses a small subset of sequenced reads (0.1–0.5× coverage) [39] as input, providing a fast and accurate way to compare two or more datasets.

Raw Illumina datasets from B- (0B) and B+ (2B) genomes [10], available at SaciBase, were used as input for the RepeatExplorer pipeline. RepeatExplorer provides a set of helper scripts to prepare the data for clustering. Reads were quality filtered based on default parameters of “paired_fastq_filtering.R” and a random sample of five million reads for each genome comprising approximately 0.5× genome coverage was selected. Finally, graph-based clustering was applied for de novo repeat identification and comparative analysis [38,39] using the RepeatExplorer pipeline with the default parameters and developer recommendations [40]. Clusters from RepeatExplorer accumulate reads that come from the two datasets and represent a common element. Thus, each cluster has an element (or similar elements) with a specific number of associated reads proportion. The results from clustering were visually inspected with respect to their graphic composition, which indicates the type of repeat and proportion of reads from each genome. We manually chose clusters with the highest content of B+ (2B) reads compared to B- (0B) reads for the later steps. The longest contigs from the selected clusters were annotated with RepeatMasker and Basic Local Alignment Search Tool (BLAST) using the command line and default parameters. To solve the eventual ambiguities in the annotation, priority was given to RepeatMasker with a joint search for conversed domains via BLAST- Conserved Domains Database (CDD); the identification of TE-related proteins was key to the classification of the assembled contigs. We used the annotated contigs found by RepeatExplorer to design primers for probe construction for fluorescence in situ hybridization and qPCR validation (see next sections).

To further characterize the repetitive content of the *A. latifasciata* B chromosome, we performed a coverage ratio analysis on alignments of the six sequenced samples (Supplementary Table S2). With this methodology, we could analyze the TE expansion on the B chromosome at individual loci. A similar approach was previously used in order to find B chromosome blocks [8,10]. The coverage ratio analysis was done as follows. Raw Illumina reads were filtered to eliminate adapters and bacterial contaminants. We created a library of adapters and bacterial genomes and aligned the reads against this library. We used Bowtie2 2.1 [41] with the “--very-fast-local” parameter, thus excluding reads similar to the sequences in the contaminant/adapter library. This protocol created a contaminant-free dataset. We checked the read quality distribution with fastQC 0.10.1 [42] and performed filtering with the FASTX-toolkit 0.0.13 [43]. We used a Phred score of 28 over 80% of the read as a quality cut-off. We then applied Pairfq 0.11 [44] to restore paired-end reads. Filtered reads were aligned with Bowtie2 against the *A. latifasciata* reference genome (see the Repeat identification and landscape pipeline section) using the “--very-sensitive” parameter. Alignment statistics from BAM files were extracted with Qualimap 2.2 [45].

Binary Alignment Map (BAM) files were used to extract coverage values. We used previously generated RepeatMasker results (see the Repeat identification and landscape pipeline section) to extract coverage for only the TE regions. Since our analysis was focused on TEs, repetitive elements with the “Simple_repeat” and “Low_complexity” traits were excluded from the annotation files. To extract coverage information from BAM files, we used bedtools 2.25 [46]. The single-copy gene hypoxanthine phosphoribosyltransferase (*HPRT*) was used as a cut-off for low-coverage regions (see details in Supplementary Methods). The average coverage for each TE interval was calculated using custom bash and python scripts. We used the average coverage of each interval to calculate the coverage ratios between the different genomes, always using the M1-0B sample as reference (Supplementary Table S3; Supplementary Methods). The ratios between *HPRT* coverages (Supplementary Table S4) were used to normalize the different read coverages among the samples.

### 2.4. Transcriptome Analysis

We had access to 36 previously sequenced messenger RNA (mRNA) libraries (available at SaciBase) from the muscle, gonads, and brain of B- (0B) and B+ (1B) males and females [47]. They were sequenced on an Illumina HiSeq 2000 (Illumina, San Diego, CA, USA) and include approximately 30 million reads each. The data are freely available at SaciBase. Reads were filtered to remove adapters and contaminants, similarly to the genomic preprocessing. They were quality filtered to maintain reads with at least a Phred score of 28 over 80% of the read. Filtered reads were submitted to RepEnrich 1.2 [48], which determines the enrichment of transcripts in the assembled genome using repeat coordinates as reference. RepEnrich returns a count table from each element and evaluates the repetitive nature of these elements; the results can be used for expression estimation within a tissue or for differential expression. We followed the developer’s tutorial to perform these analyses [49]. To find TE expression values, we used the Bioconductor package edgeR 3.4.2, and the count table was produced by RepEnrich. We calculated TE expression within each specific B- tissue to evaluate whether TE expression was present. To find the expression in a particular tissue, we used edgeR to calculate the RPKM (reads per kilobase per million mapped reads) values of each B- tissue. We separated the male and female gonads in the analysis, as they are morphologically and functionally different tissues. Later, the differential expression levels between B- and B+ samples across tissues were evaluated based on the recommendations of the RepEnrich and edgeR developers, using a generalized linear model (GLM) statistical model. We calculated the log2 fold change and false discovery rate (FDR) for each tissue and compared B- and B+ values within tissues. Male and female gonads were analyzed separately. To consider an element as differentially expressed, we used a log2 fold-change cut-off of 1.2 and an FDR cut-off of 0.05.

### 2.5. Experimental Validation of TEs

Total DNA was extracted with the Qiagen DNeasy^®^ Blood & Tissue Kit (Qiagen, Hilden, Germany) using the fish caudal fin. Primers were designed based on annotated contigs from selected RepeatExplorer clusters (Supplementary Tables S5 and S6) using Oligo Explorer^®^ 1.2 [50], Primer-BLAST [51], and PCR Primer Stats [52]. Amplicons produced by conventional PCR were sequenced using the Sanger method to confirm the sequences found by RepeatExplorer. After confirmation, sets of FISH probes were PCR-labeled with biotin-dUTP.

Mitotic chromosome preparations were performed according to [53] with modifications. Kidney tissue was dilacerated with forceps and a syringe, placed in a Potassium chloride (KCL) 0.075 M solution and incubated for 30 min. The cell suspension was fixed with a 3:1 methanol:acetic acid mixture following three washes and 900 rpm centrifugations (10 min each), discarding the supernatant at the end of each centrifugation. The slides with the chromosomes were stained with Giemsa, and the metaphases were visualized under a light microscope.

Fluorescence in situ hybridization (FISH) was performed on the chromosome squash preparations according to the protocol of [54] with modifications. Slides were dehydrated with 70% ethanol, pretreated with RNAse A (37 °C for 20 min) and pepsin/HCl (3 min), washed (3 times 2× saline sodium citrate (SSC) for 2 min each), dehydrated (2× 70% ethanol for 2 min; 2× 90% ethanol for 2 min; 1× 100% ethanol for 4 min) and dried (1 h at 60 °C). For denaturation, formamide (70%) was applied to slides for 37 s at 65 °C and immediately dehydrated (2× 70% ethanol for 2 min; 2× 90% ethanol for 2 min; 1× 100% ethanol for 4 min). Overnight hybridization was performed with the previously described PCR-labeled probes. The slides were washed (42 °C formamide (50%) for 5 min; 2 times 2× SSC for 5 min; 2 times 4× SSC/Tween for 5 min) and then detected with fluorescein isothiocyanate (FITC) (0.2 μL FITC in 100 μL 4× SSC/Tween at 37 °C for 30 min). A final wash was conducted (3 times 4× SSC/Tween for 3 min), and the slides were stained with 4′,6-diamidino-2-phenylindole (DAPI). Slides were visualized on a BX61 Olympus microscope (Olympus, Tokyo, Japan), and the hybridized metaphases were registered under an Olympus DP71 digital camera coupled to the microscope. The metaphase images were cropped using Adobe Photoshop CS2.

Quantitative real-time PCR (qPCR) of genomic DNA was performed on StepOnePlus™ Real-Time PCR equipment from Applied Biosystems, using the GoTaq^®^ qPCR Master Mix kit (Promega, Madison, USA). Cycling conditions were 95 °C for 10 min; 40 cycles of denaturation at 95 °C for 15 s and annealing/extension at 60 °C for 60 s; and 1 final cycle of dissociation at 95 °C. The results were used to calculate the gene dosage ratio (GDR) using the 2^−ΔCT^ method [55], by relative quantification with respect to the autosomal single copy gene *HPRT* on an Excel spreadsheet.

## 3. Results

### 3.1. DNA Transposable Elements Are the Most Represented Class in the A. latifasciata Genome

**Figure 1:**
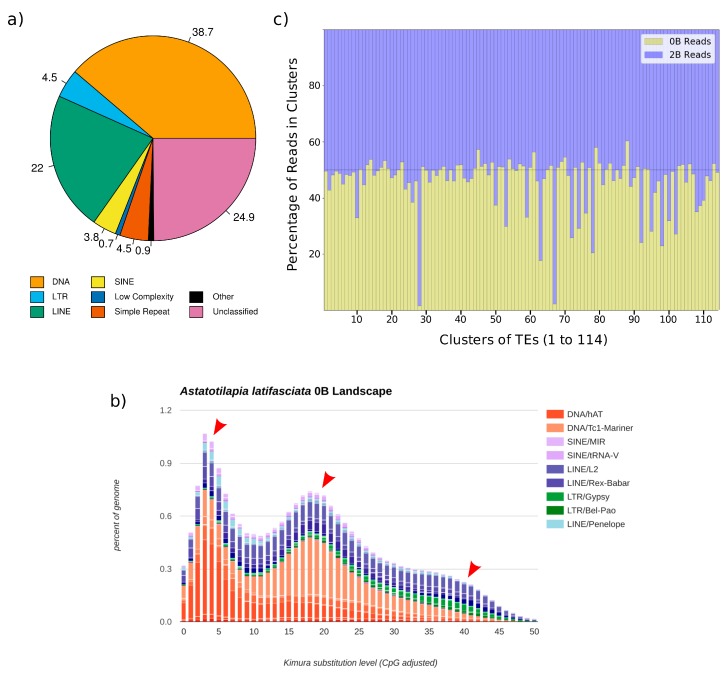
Genomic characteristics of repeated DNAs in the *Astatotilapia latifasciata* genome. (**a**) Percentage of each type of repetitive sequence in the total repetitive portion detected by RepeatMasker on the B- genome. Numbers in this figure represent the percentage of each repeat group compared to the total repetitive content, rather than their percentage in the genome. DNA transposons and long interspersed nuclear element (LINEs) dominate the repetitive content. Unclassified sequences comprise mostly multigenic families and gene clusters. (**b**) Repeat landscape of the *A. latifasciata* genome. For each element, the graph shows the sequence divergence from its consensus (*x*-axis) in relation to the number of copies on the genome (*y*-axis). Peaks represent insertion waves (red arrows) of elements into the genome. Elements with older insertion waves are shown on the right side of the graph, while newer insertions are depicted on the left side. Different colors show distinct element types, as described at the right. For a detailed and interactive version of the graph, please refer to Supplemental file 2. (**c**) Comparative analysis of repeats on the B- (0B) and B+ (2B) genomes via RepeatExplorer. Each column represents 100% of the reads in a cluster; the read proportions from the B- (0B) and B+ (2B) genomes are shown in yellow and blue, respectively. Clusters with higher proportions of B+ (2B) reads (in blue) are shown by an expansion of the blue color into the lower part of the graph (yellow). LINE: Long interspersed nuclear element; LTR: Long terminal repeat; SINE: Short interspersed nuclear element; TE: Transposable element.

We first focused on the repetitive content in the B- genome and detected similar patterns of TE composition among *A. latifasciata* and other cichlids [51,52]. An estimation of TE copy number and percentage on the B- genome, here considered the canonical genome, was obtained with RepeatMasker (Figure 1a, Supplementary Table S7, and Supplementary file 1). The *A. latifasciata* genome contains 28.41% repetitive DNA, the majority of which consists of TEs. DNA transposons are the most represented category, followed by long interspersed nuclear element (LINE), LTR, and short interspersed nuclear element (SINE) retrotransposons. In total, retrotransposons (LTR, LINE, and SINE) represent 354,288 copies, while 464,043 copies of DNA transposons are present. Among the retroelements, LINEs had both higher copy number and more bases masked on the genome. SINEs are present in higher number than LTRs, but LTRs have more bases masked on the genome, probably due to their larger size. We found slightly over 7% unclassified elements in the genome.

Tc1-Mariner (4.37% of the genome) and hAT-Ac (1.77% of the genome) are the most highly represented DNA elements. Together, they represent over 50% of the DNA transposons in the *A. latifasciata* genome. A high diversity of other DNA elements is present, but each of them represents only a small percentage of the total. The predominant LINE is L2 (2.44%), but the element Rex (1.06% of the genome) is also represented. All major classes of LTR are found in the genome, with the Gypsy (0.61%) and Pao (Bel/Pao, 0.21%) classes being the most abundant. Among SINEs, a high variation in element copy number is present, and families derived from transfer RNA (tRNA) are the most abundant.

These findings show that *A. latifasciata* has a similar transposable element composition to those of other available cichlid fish genomes (see Discussion section).

### 3.2. Three Main Bursts of DNA Element Insertion during Evolution Explain Their Genomic Abundance

Using the assembled B- genome and RepeatMasker results, a repeat landscape was constructed (Figure 1b, and Supplementary file 2 for an interactive version). It indicates the relative waves of insertion of specific families in the genome and can show bursts of insertion events during the evolution of the species, where a rapid increase in repeat copy number occurred. From these bursts, we can infer mobilization events in the evolution of the genome as well as putative TE activity represented by newer insertions [56]. Three main burst events were found in *A. latifasciata*’s evolution. In an older burst, a small accumulation of retrotransposons, both LTRs (Bel/Pao, Gypsy, and ERV) and LINEs (Rex-Babar and L2) occurred. The second burst of TEs had DNA elements as its major component. Tc1-Mariner showed the greatest increase in copy number during this period. The third and most recent burst of TE insertions was due to the accumulation of hAT DNA elements. Although hAT copy number began to increase in the second insertion wave, the third insertion wave involved predominantly hAT elements. As a trend, we see DNA elements dominating the landscape of TE insertions during *A. latifasciata* genome evolution. Retroelements show signs of constant mobilization; for example, L2 and Rex, which seem to have both younger and older copies, are represented by a constant appearance in the landscape.

### 3.3. Accumulation of TEs in the B Chromosome of A. latifasciata

Repetitive element accumulation is a hallmark of B chromosomes. Understanding the TE compositions of Bs can assist in the determination of their origins and possible effects in the cell. To establish whether an accumulation of TEs is present in the B chromosome of *A. latifasciata*, we performed a comparative analysis using RepeatExplorer and coverage ratios. The RepeatExplorer pipeline yielded 114 clusters (Supplementary file 3) with approximately 75% of the reads in clusters and 25% in singlets. For each cluster, we visually compared the number of reads from the B- (0B) and B+ (2B) datasets. Clusters having higher proportions of B+ (2B) reads (i.e., the number of 2B reads was much higher than the number of 0B reads) were selected for manual inspection and subsequent analysis (Figure 1c, Table 1, Supplementary file 3). From a total of 20 analyzed clusters, four were chosen for FISH probe construction (see next section for validation of results); clusters with better contig annotations and genomic relevance were selected. The clusters with higher proportions of 2B reads were clusters 28 and 67, annotated as Bel/Pao and Gypsy, respectively. This result indicates an expansion of those TEs on the extra chromosome. Other clusters selected for FISH were cluster 63 (hAT element) and cluster 74 (L2 element).

Although RepeatExplorer returned different expanded elements on the B chromosome, we decided to further analyze these elements to obtain a more detailed picture of B chromosome TE composition. In this way, we validated the RepeatExplorer results by bioinformatics and built a database of putative expanded elements with their respective loci. We performed a coverage ratio analysis on the several NGS datasets available (0B, 1B, and 2B, males and females; Supplementary Tables S1 and S2).

The coverage ratios showed that several elements were expanded on the B chromosome, consistent with the RepeatExplorer results (Table 2; Supplementary file 4). In general, the repeat class with the most accumulation on B was DNA transposons, followed by LINEs and LTRs. A large number of unclassified sequences was also found on B, showing the mobility of diverse sequences to the supernumerary element.

Examples of major TEs expanded on the B chromosome, as shown by coverage ratio analysis, include Gypsy-188_DR-I (Gypsy), AlRepB-26 (L2), Bel32-I_DR (Bel/Pao), Mariner-N13_DR (Tc1-Mariner), and AlRepB-738 (hAT-Ac) (Table 1 and Table 2). RepeatExplorer also found elements from these families, and some of them were validated by qPCR, sequencing, and FISH (see next section). The copy number of these elements varies with the number of Bs in the genome; a B+ (2B) individual has accumulated more copies than a B+ (1B) individual has. Several other TEs also follow this pattern (Table 2).

Not only do our results point to the accumulation of TEs in the B chromosome, but the data also show different trends in the accumulation of elements by sex or by B presence and sex. For example, the Maui element (member of L2 family) is expanded in females and B chromosome carriers. Additionally, the element AlRepC-927 (hAT family) is absent in the B+ (2B) individual, which may indicate a recent mobilization or a population-related bias. Although an individual analysis of each element lies outside the scope of this work, such examples show the plasticity and mobility that TEs can achieve, especially considering the presence of a B chromosome.

### 3.4. B Chromosome TE Accumulation Is Validated by Cytogenetics and Molecular Techniques

**Figure 2:**
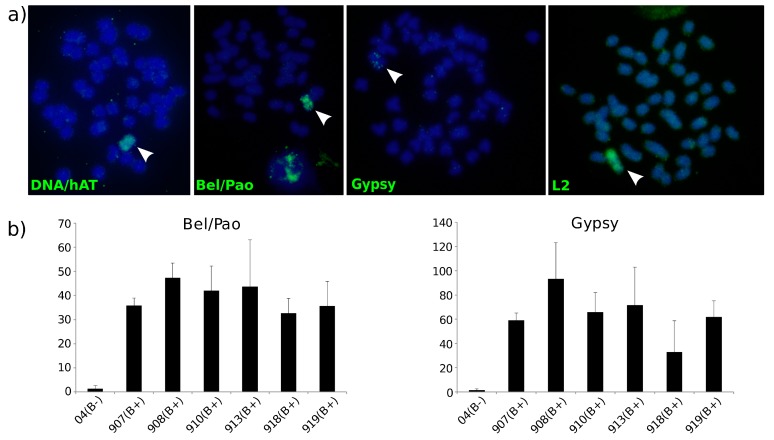
Distribution and copy number variation of repetitive DNAs in the *A. latifasciata* genome. (**a**) Fluorescent in situ hybridization mapping of four selected elements (DNA/hAT, Bel/Pao, Gypsy, and L2) on *A. latifasciata* metaphasic chromosomes, from a B+ specimen. Probes were PCR-labeled by biotin-dUTP, and the signal was detected by fluorescein isothiocyanate (FITC, green). Red arrowheads indicate the B chromosomes. The signal intensity is higher in the B chromosome than in the A complement. (**b**) qPCR of the Bel/Pao and Gypsy elements detected by the RepeatExplorer pipeline. Sample number 04 had no B chromosome (B-), while the others had at least one B (B+). The data show that B+ individuals had higher copy numbers than B- individuals, corroborating the previous results. GDR: Gene dosage ratio.

Based on the bioinformatics analysis, we expected a higher number of TE copies in the B chromosome than in the A complement. Therefore, we sought to validate our bioinformatics findings concerning B chromosome TE composition. Sequences detected by RepeatExplorer were used to design a custom set of primers to validate the elements expanded in the B chromosome of *A. latifasciata* (Supplementary Tables S5 and S6). Custom probes were obtained by PCR and hybridized on mitotic chromosome preparations (Supplementary Table S5). Fluorescence in situ hybridization analysis revealed intense hybridization signals, mostly in the metacentric B chromosome (Figure 2a). As predicted by the bioinformatics pipeline, the signals from the custom probes were concentrated in the B chromosome, although they also had a diffuse presence in the A complement.

Quantitative real-time PCR was used to quantify the sequences in relation to the normalizer, the single-copy gene *HPRT*. The Bel/Pao and Gypsy elements were chosen due their abundance in the B chromosome (Supplementary Table S6). Relative quantification showed higher copy numbers of these elements in B+ individuals (Figure 2b). All B+ samples showed higher GDRs than the B- samples (sample 04). Thus, the results show that B+ individuals had higher element copy numbers than did B- individuals. The data from qPCR agreed with the results of RepeatExplorer and FISH, which demonstrated the accumulation of certain elements in the B chromosome (in this case, Bel/Pao and Gypsy).

### 3.5. TE Transcription across Tissues and Higher Expression in B Chromosome Samples

Based on the TE structural analysis of the B- and B+ genomes, we sought to establish the transcription levels of TEs in both conditions. According to the repeat landscape, some elements show signs of recent mobilization, and our first aim was to determine whether these elements are transcribed. The B- RPKM expression values show that many TEs are transcribed in various *A. latifasciata* tissues (Supplementary Figure S1; Supplementary Table S7; Supplementary file 5). All major families are expressed, with high variability among the individual elements. Although not suitable for comparisons between tissues, these RPKM values reveal that TE transcription occurs in the *A. latifasciata* genome. In fact, elements with recent insertion waves, which are thus putatively active, are expressed in different tissues. Tc1-Mariner and hAT have the highest expression levels among DNA transposons. L2, RTE-BovB, Rex, L1, Penelope, and MIR have high expression among retrotransposons. Such elements exemplify the relation between the repeat landscape and activity in the genome. The expression of each family tended to be constant among the tissues.

**Figure 3:**
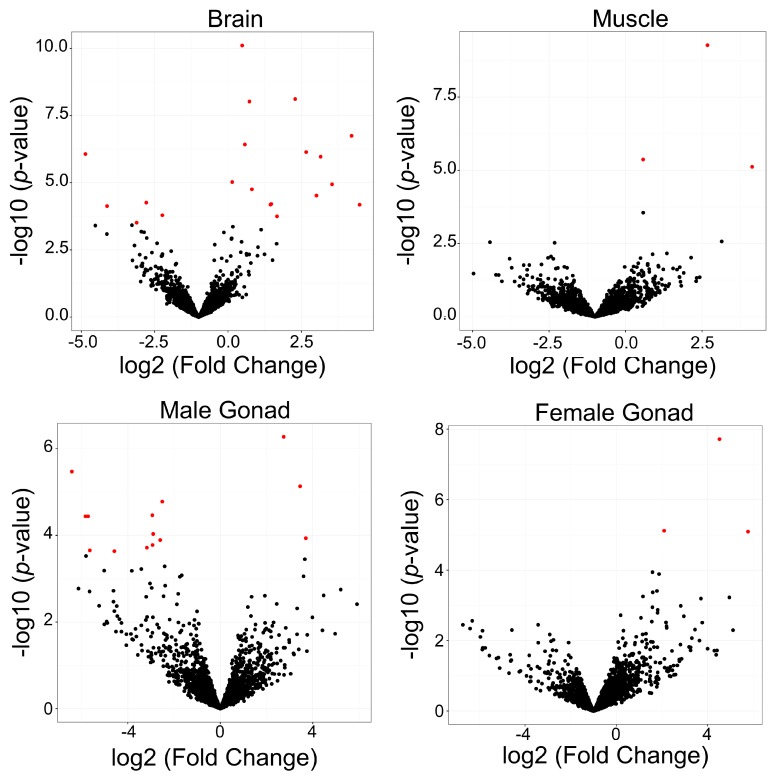
Volcano plots showing differential expression between B- and B+ individuals in brain, muscle, and male and female gonads. Red points indicate elements with differential expression above the threshold (log2 fold-change 1.2 and false discovery rate (FDR) <5%). Upregulated repeats in the B+ genomes are represented on the right side, and downregulated repeats in the B+ genomes are represented on the left side of each figure.

Next, we examined whether elements in the B chromosome had different expression levels than those in the rest of the genome. Such a difference would show the putative activity of elements present in the B chromosome compared to those in the A complement. All tissues showed differential expression of repetitive elements between the B+ (1B) and B- (0B) genomes (Figure 3; Table 3; Supplementary file 6). A high variability was found in the families expressed in different tissues, but DNA transposons showed differential expression in all of them. In brain, 15 upregulated and 5 downregulated elements were found. Retroelements dominated the differential expression; 14 out of 20 differentially expressed sequences were retroelements. In muscle, only three elements showed differential expression, and all were upregulated in the B+ tissue. Excluding an unclassified element, the other two were DNA transposons. In male gonads, we detected 14 differentially expressed elements, with only 3 upregulated in the B+ genome. In female gonads, only three elements showed differential expression, and all were upregulated in the B+ genome. Our results indicate that, with the exception of male gonads, a trend toward the upregulation of elements in B+ tissues is present.

Most elements with differential expression between the B- and B+ genomes were not accumulated in the B chromosome. Exceptions existed, such as the element AlRepB-358 (hAT family), which had differential expression in B+ muscle and high copy number in 1B individuals, according to the coverage ratio analysis.

The only two elements with differential expression in two tissues (brain and muscle) were Mariner-1_SP and AlRepC-299. A BLAST of AlRepC-299 revealed 98% similarity with sequences from the *V2R* gene cluster of the cichlid *Haplochromis chilotes* (accession number AB780556.1) and 97% identity with sequences from *Oreochromis niloticus* (also a cichlid) in a region with no annotation but close to pseudogenes (accession number AB270897.1).

## 4. Discussion

### 4.1. Genomic Organization of TEs in A. latifasciata

The primary aim of our work was to characterize the repetitive content of the reference B- genome of *A. latifasciata* and show its relation to those of other cichlid fishes. DNA transposons were the major TE class found, while retroelements represented a smaller fraction. This composition is similar to those of other teleost fishes, including other African cichlids. In general, teleost fishes show one of the greatest diversities of TEs among vertebrates, with a high number of families populating the genome [57,58]. Among DNA transposons, Tc1-Mariner and hAT (class II TEs) are represented at higher percentages in the genome of *A. latifasciata*. This result is similar to those in other African cichlids, where class II elements are responsible for approximately 10% of repetitive content, a number also observed in *A. latifasciata*. L2 (a LINE retroelement) also predominates in the genome and, with Tc1-Mariner, has the highest genome percentage in both *A. latifasciata* and other cichlid fishes [57,59].

Next, we constructed a repeat landscape that shows several waves of TE insertion into the genome. We found that LTRs such as Gypsy and Bel/Pao entered the genome in its early evolutionary stages. DNA transposons such as Tc1-Mariner had later insertions and are accompanied by large increases in copy number. Similar landscapes are found in other cichlid fishes [59], and together with the TE content results, they support the idea of TE mobilization events in a common ancestor of the group. In general, TE families with high copy numbers in the genome of *A. latifasciata* show prominent insertion waves and expansions in the B chromosome. Elements with higher copy numbers seem to dominate B genome content, probably due to the low selective pressure on B. These findings demonstrate the ability of the B chromosome to act as a repository for sequences that can propagate over time.

The insertions of Penelope, Rex-Babar, L2, Tc1-Mariner, and hAT elements may indicate recent activity. In fact, these families have several elements with high expression in the analyzed tissues, and thus, some copies may still be active and populating new loci. Furthermore, some families are constant on the landscape, which indicates active mobilization during the evolution of the genome. Our data show a landmark of TEs: their activity is variable over time, with bursts following purification cycles [60]. Additionally, the correlation of transcription with the repeat landscape can help delimit elements for future studies of repeat mobilization and function.

Among the most highly transcribed elements, several have high copy numbers in the genome. Although transcription is not directly correlated to mobilization events, some expressed elements are related to recent insertion waves; some sequences even show evidence of open reading frames (ORFs) capable of expressing protein-coding sequences (data not shown). Considering that transposition events are a major cause of genomic instability and rearrangement [61], we should consider that, although transcribed, these copies might have lost the capacity to mobilize.

Environmental conditions can alter TE activity, change their copy number, and cause large genome alterations, especially through epigenetic silencing modifications. Posttranscriptional alterations such as small interfering (siRNA) can silence TEs and limit their copy number increase, abolishing the correlation between TE transcription and transposition events [60,62,63]. Even with several transcribed elements in the genome, posttranscriptional silencing can limit their transposition and copy number alteration. At this point, even with evidence of recent mobilization events and element transcription, we speculate that tight control is exerted on TEs.

### 4.2. Organization and Transcription of TEs in the B Chromosome

After examining the TE composition and expression in the B- genome, we were interested in searching for these characteristics in the B chromosome. For the first comparison between the B- and B+ genome datasets, we used RepeatExplorer, which is a de novo method of element identification and assembly [38,58] that can be used for comparative quantification between two or more datasets [64]. RepeatExplorer has the advantage of using raw Illumina reads with very low coverage; we selected this method for FISH probe construction and to test our hypothesis. Coverage information from the clusters obtained by RepeatExplorer is indicative of expanded elements in a particular dataset [39]. According to [38], the number of reads in a cluster is proportional to the quantity of that element on the genome. Here, we used a combination of methods (RepeatExplorer and coverage ratios) to give a broad picture of *A. latifasciata* B chromosome TE composition.

With RepeatExplorer, we could identify and validate several expanded elements on the B chromosome. All FISH-mapped elements showed similar patterns of hybridization, demonstrating a high number of copies on the B chromosome and thus corroborating the clustering data. We chose to perform a second validation with qPCR with the two elements most present in the B chromosome. Gypsy and Bel/Pao were selected for their abundance on the B chromosome compared to that on the A complement. Both analyses validated the B expansion of these specific TEs.

After our finding of expanded elements on the B chromosome of *A. latifasciata*, our aim was to specify the repeat families present and their loci; for that, we used a coverage ratio approach. The data from the coverage ratio analysis showed the diversity of TE composition on the B chromosome of the species and corroborated the results of RepeatExplorer. The elements detected by RepeatExplorer showed more coverage in B+ genomes. The results of the coverage ratios reflect the sum of all copies that are at least duplicated in the B chromosome. We selected this conservative threshold due to the inherent variation in repeat copy numbers. For some elements, 1B and 2B individuals had a two-times difference in coverage values, showing the effect of the number of B chromosomes in a species. Since this pattern was not present in all elements, it also indicates the variability of B sequences. Fluctuations in B block copy number are present even among siblings, as found by [29] in *Metriaclima lombardoi*, and our results corroborate these findings. Our sequencing data comes from different populations and is also prone to variation among individuals, which was evidenced by coverage analysis and qPCR.

Another characteristic detected by RepeatExplorer and by the coverage ratio analysis is the number of other types of sequences in the B chromosome. The *V2R* gene sequence was found in some contigs assembled by RepeatExplorer, along with *HOX* and *SOX* gene fragments. Considering the quantity of retroelements expanded on the B, these elements may have transferred a number of sequences during their activity cycles, as they are facilitators of sequence mobilization [65]. Retroelements such as LINEs have weak poly-A signals and are good candidates to facilitate the mobilization of adjacent sequences. Furthermore, enzymes can also incorporate non-TE mRNAs [66,67], leading to the inclusion of different types of sequences in the B chromosome. In general, TEs take advantage of the low selective pressure on the B chromosome for their insertion and perpetuation [3]. The combination of low selective pressure and drive (a B chromosome characteristic) allows repeats to expand and increase copy number in the B chromosome [9,10]. Such expansion or contraction of sequences in Bs is a hallmark of supernumeraries in diverse species [64], and is now presented here for *A. latifasciata*. Our results support the findings from other B chromosomes, which also show remarkable sequence expansions.

In addition to the analysis of TE expression in the B- genome, another goal of this study was to find signs of TE expression in the B chromosome of this species. Our data show evidence of the transcription of B chromosome sequences, although only a few TEs demonstrated differential expression between the B- and B+ genomes. This result points to a relatively stable B chromosome and is an important finding of our study. Moreover, differentially expressed elements expanded on the B chromosome usually had coverage differences smaller than those of highly expanded ones, such as Gypsy-188 and Bel32. Therefore, the majority of expanded elements in B+ genomes did not show differential expression between B- and B+ individuals. Expanded elements may have been present in the early stages of B chromosome formation, and we hypothesize that silencing mechanisms target them. If not silenced, elements with high copy numbers in the B chromosome could generate high genomic instability [60]. In general, increases in repeat copy number cause their transcriptional reduction through silencing mechanisms [68,69]. However, elements with high copy number can have high expression, as in the hAT family in maize [70]. Expression levels are also influenced by the number of B chromosomes in the cell; a higher number of Bs is associated with higher differential expression [25]. Our data are based on 1B samples, and therefore, small changes in differential expression from the B sequences may be difficult to detect. Our findings also point to a general trend of opposite TE expression in the gonads. Several elements are downregulated in males, while some are upregulated in females. We speculate that this DE among males and females could benefit the B chromosome drive exclusive of female meiosis [71].

The expression of repetitive elements on B chromosomes is highly dependent on the species and the location of the repeat in the supernumerary element. Expression is also related to the TE family. In rye, the elements expressed on the B chromosome are correlated to the presence of high copy number in the genome and even modulate the transcription of A-complement copies [6]. Differential expression among 0B and 4B rye individuals revealed a high quantity of expressed B repeats together with several gene fragments [26]. Gypsy and Mariner elements are expanded and expressed on the B chromosome of the grasshopper *Eyprepocnemis plorans*, with a predisposition to euchromatic regions [72]. Considering that methylation patterns vary on each B chromosome, their transcription levels may be altered. DNA methylation inactivates B chromosomes, reducing their effects on the cell [73]. It is plausible that the high-copy number elements in the B+ genome of *A. latifasciata* have their transcription regulated to avoid disruptive interference in the physiology of the cell. The B chromosome of *A. latifasciata* has the potential to generate noncoding RNA regulatory sequences [74], and therefore the regulation of this extra chromosome could help to stabilize the genome.

## 5. Conclusions

Research on TEs, especially those on B chromosomes, brings technical and analytical challenges, but understanding their organization in the genome can shed light on intrinsic mechanisms of gene and genome regulation. In this regard, this study is an important step to clarify B chromosome organization and structure. We found several expanded elements on the B chromosome of *A. latifasciata* and confirmed them through high-throughput sequencing, bioinformatics, FISH mapping, and qPCR quantification. We also described a general landscape of repeat copy number, relative insertion times in the genome, and transcription levels.

The influence of TEs on shaping the genome is clear, and studying their role in the formation and perpetuation of B chromosomes requires different approaches, such as the investigation of the transcription levels of messenger RNAs and noncoding RNAs. We found evidence that expansion of TEs on the B chromosome can be followed by the evolution of regulatory mechanisms that control TE activity. A deeper understanding of the epigenetic mechanisms acting on the accessory chromosome will also be necessary to determine the level of influence of the discovered elements on B.

## Figures and Tables

**Table 1:** Information of clusters with higher proportions of 2B reads selected for fluorescence in situ hybridization (FISH) probe construction.

Cluster	0B Reads	2B Reads	Annotation	Repeat Class
28	359	23,389	Bel/Pao	Retro/LTR
63	2939	13,563	DNA/hAT	DNA
67	351	15,603	Gypsy	Retro/LTR
74	4575	11,117	L2	Retro/LINE

Retro: Retrotransposon; DNA: DNA transposon; LTR: Long terminal repeat retrotransposon; LINE: Long interspersed nuclear element.

**Table 2:** Coverage ratio analysis of the six sequenced individuals. Values were normalized to the M1-0B genome (B- male). Values show the number of extra copies in a sample compared to that in the reference. Numbers are the sum of all loci expanded on the B chromosome.

Element	Class	Family	F1-0B/M1-0B	F2-1B/M1-0B	M2-1B/M1-0B	M3-1B/M1-0B	M4-2B/M1-0B
Gypsy-188_DR-I	LTR	Gypsy	0.00	357.90	478.75	505.95	977.98
AlRepB-26	LINE	L2	2.07	454.47	438.12	454.37	976.85
BEL32-I_DR	LTR	Pao	11.24	276.16	324.04	343.45	731.48
Gypsy-23_GA-I	LTR	Gypsy	0.00	311.83	336.77	360.23	709.62
Mariner-N13_DR	DNA	TcMar-Tc1	17.81	276.37	294.65	312.57	648.31
AlRepB-738	DNA	hAT-Ac	23.67	320.79	356.62	382.23	631.88
BEL32-LTR_DR	LTR	Pao	5.46	215.99	278.12	309.23	593.38
Maui	LINE	L2	777.82	916.34	626.59	665.77	312.38
AlRepC-927	DNA	hAT-Ac	253.98	250.69	96.81	92.91	0.00
AlRepB-157	Satellite	Satellite	545.89	537.41	39.69	23.75	44.40
TZSAT	Satellite	Satellite	181.04	177.25	4.73	4.52	6.86

M: Male; F: Female; 0B: Absence of B chromosomes; 1B: Presence of one B chromosome; 2B: Presence of two B chromosomes.

**Table 3:** Repetitive elements with differential expression between B- and B+ individuals. B expansion represents how many more copies an individual had than the reference M1-0B. RC, rolling circle TE; srpRNA, signal recognition particle RNA.

Element	Superfamily	Class	Fold-Change	B Expansion
**Brain**				
AlRepD-1119	L2	LINE	1.4829	22/88/115/126/187
CR1-28_HM	CR1	LINE	3.2887	No
REX1-3_XT	Rex-Babar	LINE	1.7297	No
GYPSY2-I_CB	Gypsy	LTR	5.2102	No
AlRepC-299	Unknown	Unknown	1.5748	9/16/4/6/0
I-6_AAe	I	LINE	3.6624	No
L1-6_DR	L1	LINE	−3.8609	2/0/0/0/2
BovBa-1_EF	RTE-BovB	LINE	4.1560	No
AlRepD-1964	Unknown	Unknown	1.1420	24/117/121/120/160
Gypsy-34-I_DR	Gypsy	LTR	4.5448	No
AlRepD-520	Unknown	Unknown	1.8113	47/30/6/2/0
Mariner-1_SP	TcMar-Fot1	DNA	4.0150	0/0/2/5/12
ERV1-3N-EC_I-int	ERV1	LTR	2.4732	No
Gypsy-17-I_DR	Gypsy	LTR	−1.7877	2/2/2/4/16
Gypsy52-I_DR	Gypsy	LTR	5.4846	No
Tc1-2Eso	TcMar-Tc1	DNA	2.4478	No
Rex1-52_DR	Rex-Babar	LINE	−3.1220	No
Helitron-2_DR	Helitron	RC	−1.2385	No
RMER17C-int	ERVK	LTR	2.6684	No
Gypsy-20-I_DR	Gypsy	LTR	−2.1144	0/0/2/0/0
**Muscle**				
AlRepC-299	Unknown	Unknown	7.5228	9/16/4/6/0
AlRepB-358	hAT-Ac	DNA	3.1883	207/209/146/139/11
Mariner-1_SP	TcMar-Fot1	DNA	11.0098	0/0/2/5/12
**Gonad Male**				
AlRepD-4130	hAT-Ac	DNA	2.7431	37/87/51/56/31
HEROTn	R2-Hero	LINE	−6.4246	No
7SLRNA	srpRNA	srpRNA	3.4512	No
P-27_HM	P	DNA	−2.5097	No
AgaP15	P	DNA	−5.8314	No
Charlie16	hAT-Charlie	DNA	−5.7227	No
AlRepE-134	DNA	DNA	−2.9391	6/3/3/5/3
ERV-4_CPB-I	ERV1	LTR	−2.9007	No
Gypsy-10_GA-LTR	Gypsy	LTR	−2.6009	No
AlRepE-2243	Unknown	Unknown	3.6990	5/2/2/0/2
CR1-20_CQ	CR1	LINE	−2.9297	No
Dada-1_ON	Dada	DNA	−3.1755	No
Copia3-I_XT	Copia	LTR	−5.6443	No
AlRepD-3555	Unknown	Unknown	−4.5855	No
**Gonad Female**				
AlRepD-1636	Unknown	Unknown	5.5199	23/94/96/104/111
hAT-27_LCh	DNA	DNA	3.0980	No
AlRepD-4141	Unknown	Unknown	6.7706	2/0/0/0/2

Each number is the sum of all loci in the analyzed individuals (F1-0B/M1-0B, F2-1B/M1-0B, M2-1B/M1-0B, M3-1B/M1-0B, M4-2B/M1-0B).

## Supplementary Materials

The following are available online at http://www.mdpi.com/2073-4425/9/6/269/s1. Figure S1: Repetitive element transcription levels quantified by RPKM, Table S1: Summary of most important information about the investigated samples, Table S2: Samples and statistics for the six read dataset alignments against the *A. latifasciata* reference genome, Table S3: Metrics of HPRT gene coverage in the six alignments, Table S4: Ratios between HPRT coverages in reference to the M1-0B dataset, Table S5: Primers used for the construction of custom FISH probes, Table S6: Primers used for qPCR, Table S7: Copy number estimates for several elements found in the *A. latifasciata* genome, File S1: Repeat copy numbers in the *A. latifasciata* 0B genome, File S2: *A. latifasciata* 0B landscape, File S3: Clusters from RepeatExplorer, File S4: Coverage ratios normalized to the M1-0B genome as reference, File S5: RPKM values of repetitive elements from brain, muscle and male and female gonads, File S6: Differential expression values between B- and B+ in brain, muscle and male and female gonads, Supplementary Methods: Determination of cut-off for coverage regions. References [75,76,77] are cited in the supplementary materials.
