# Supplementary material for: Landscape of Transposable Elements Focusing on the B Chromosome of the Cichlid Fish Astatotilapia latifasciata
Source: Genes (Basel). 2018 May 23;9(6):269. doi: 10.3390/genes9060269 (PMC6027319; doi:10.3390/genes9060269)
Supplement: Supplementary file 1 [file genes-09-00269-s001.zip › Supplementary_file_2.html]

Repeat Landscape

Repeat Landscape


---

© RepeatMasker.org
