# Supplementary material for: Landscape of Transposable Elements Focusing on the B Chromosome of the Cichlid Fish Astatotilapia latifasciata
Source: Genes (Basel). 2018 May 23;9(6):269. doi: 10.3390/genes9060269 (PMC6027319; doi:10.3390/genes9060269)
Supplement: Supplementary file 1 [file genes-09-00269-s001.zip › Supplementary_file_3.pdf]

Supplementary file 3: clusters from RepeatExplorer.

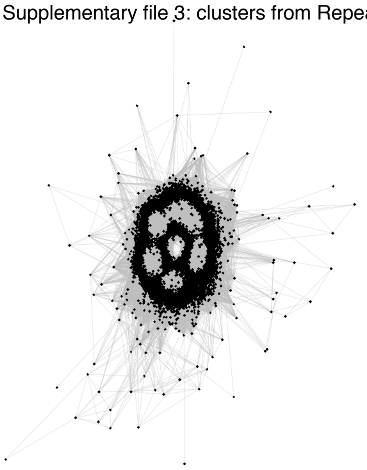

**CL1**

Number of reads: 13288
Number of pairs: 24887532
Density: 0.2819
Diameter: NA
Mean edge weigth: 125.98
Max. degree: 5056

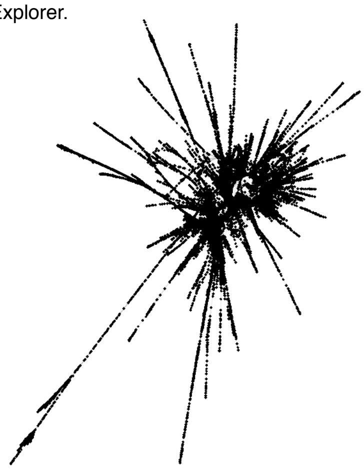

**CL2**

Number of reads: 43143
Number of pairs: 1833126
Density: 0.00197
Diameter: NA
Mean edge weigth: 134.03
Max. degree: 750

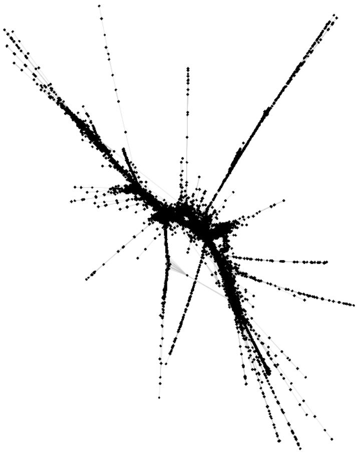

**CL3**

Number of reads: 40049
Number of pairs: 17095032
Density: 0.02132
Diameter: NA
Mean edge weigth: 118.99
Max. degree: 2608

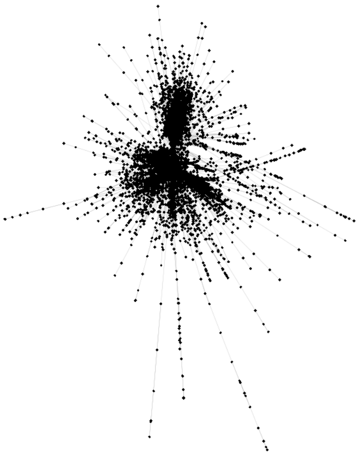

**CL4**

Number of reads: 38815
Number of pairs: 24980157
Density: 0.03316
Diameter: NA
Mean edge weigth: 114.21
Max. degree: 5347

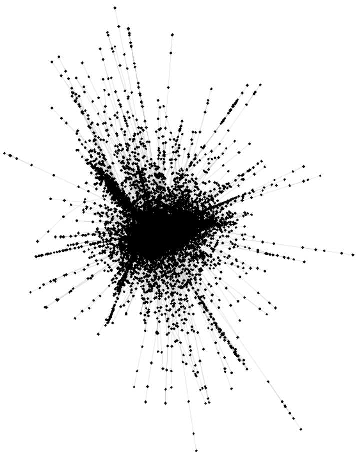

**CL5**

Number of reads: 34042
Number of pairs: 24963817
Density: 0.04308
Diameter: NA
Mean edge weigth: 118.15
Max. degree: 6939

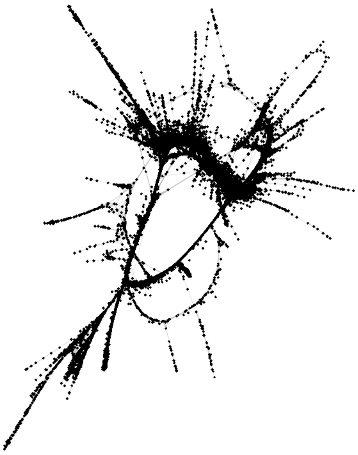

**CL6**

Number of reads: 38149
Number of pairs: 6519854
Density: 0.00896
Diameter: NA
Mean edge weigth: 136.9
Max. degree: 1349

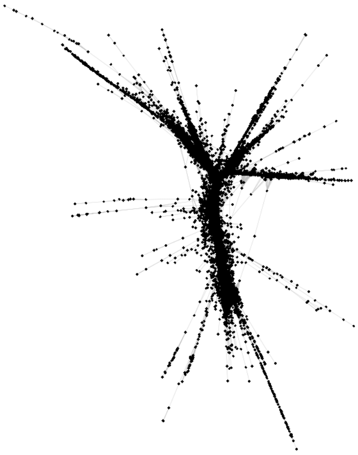

**CL7**

Number of reads: 34628
Number of pairs: 17040146
Density: 0.02842
Diameter: NA
Mean edge weigth: 118.27
Max. degree: 3382

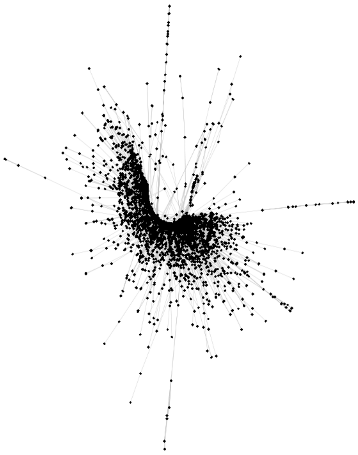

**CL8**

Number of reads: 27553
Number of pairs: 24922448
Density: 0.06566
Diameter: NA
Mean edge weigth: 134.44
Max. degree: 2955

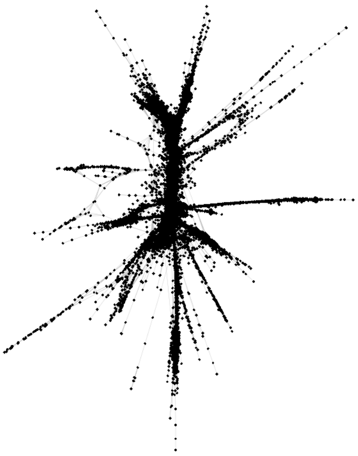

**CL9**

Number of reads: 33471
Number of pairs: 8469047
Density: 0.01512
Diameter: NA
Mean edge weigth: 121.25
Max. degree: 2160

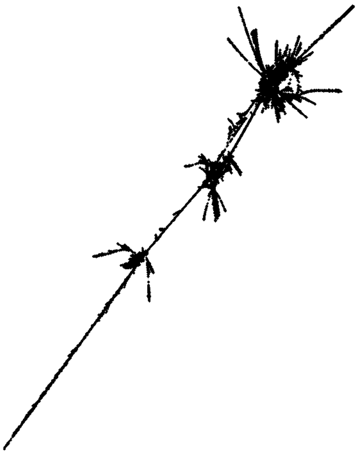

**CL10**

Number of reads: 33129
Number of pairs: 2678185
Density: 0.004881
Diameter: NA
Mean edge weigth: 138.69
Max. degree: 711

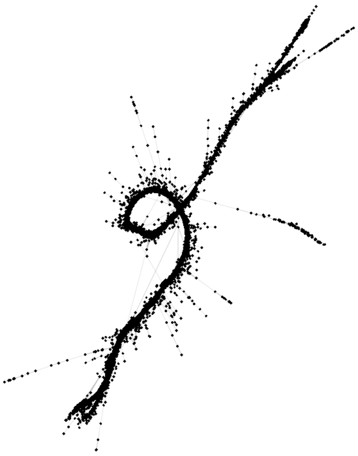

**CL11**

Number of reads: 31882
Number of pairs: 6992372
Density: 0.01376
Diameter: NA
Mean edge weigth: 132.01
Max. degree: 923

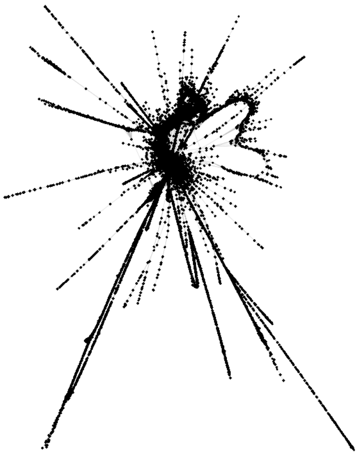

**CL12**

Number of reads: 31193
Number of pairs: 3941314
Density: 0.008102
Diameter: NA
Mean edge weigth: 109.16
Max. degree: 2562

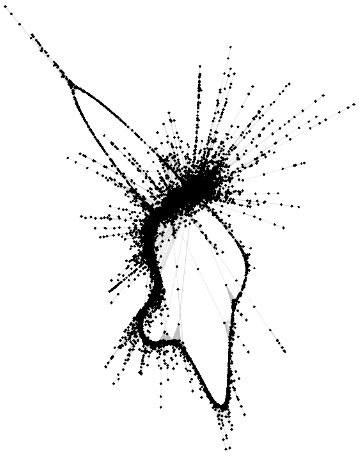

**CL13**

Number of reads: 30476
Number of pairs: 15560172
Density: 0.03351
Diameter: NA
Mean edge weigth: 120.62
Max. degree: 4244

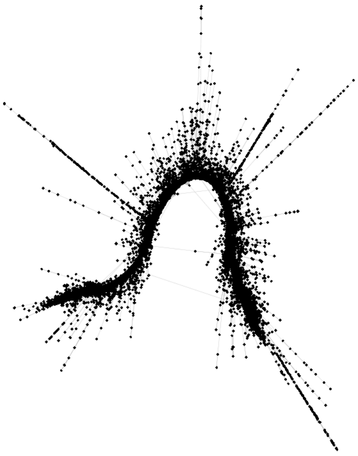

**CL14**

Number of reads: 30465
Number of pairs: 11960413
Density: 0.02577
Diameter: NA
Mean edge weigth: 135.77
Max. degree: 1569

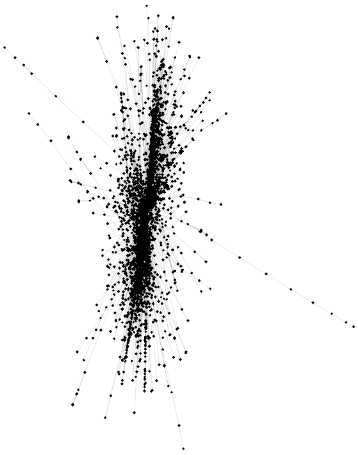

**CL15**

Number of reads: 23282
Number of pairs: 25124565
Density: 0.09271
Diameter: NA
Mean edge weigth: 133.33
Max. degree: 3677

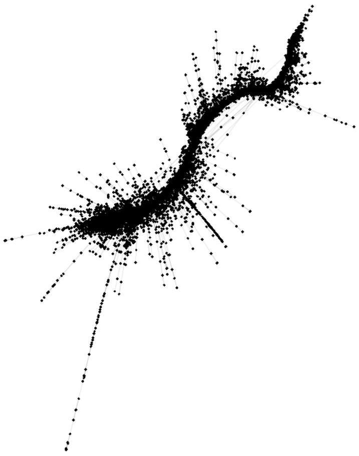

**CL16**

Number of reads: 28696
Number of pairs: 11355549
Density: 0.02758
Diameter: NA
Mean edge weigth: 130.97
Max. degree: 1770

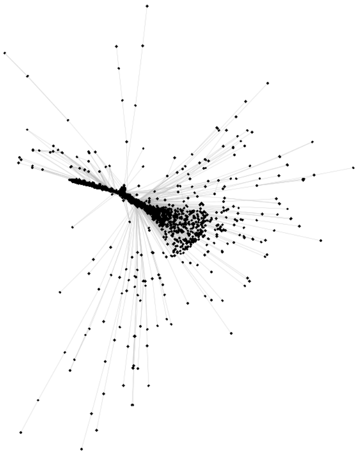

**CL17**

Number of reads: 16532
Number of pairs: 25182157
Density: 0.1843
Diameter: NA
Mean edge weigth: 140.71
Max. degree: 4994

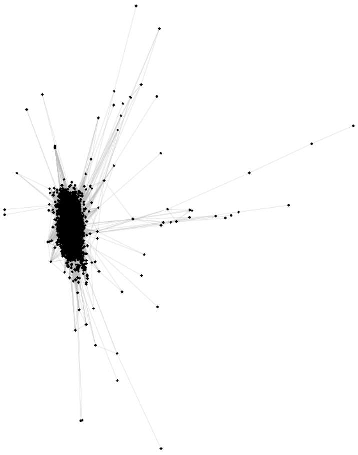

**CL18**

Number of reads: 24234
Number of pairs: 24858135
Density: 0.08466
Diameter: NA
Mean edge weigth: 107.29
Max. degree: 18895

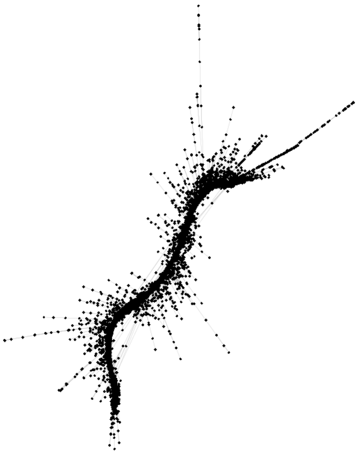

**CL19**

Number of reads: 26828
Number of pairs: 14572225
Density: 0.04049
Diameter: NA
Mean edge weigth: 136.96
Max. degree: 2056

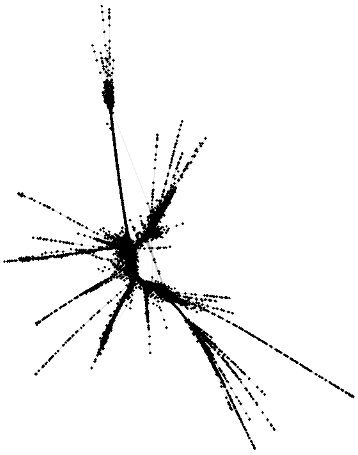

**CL20**

Number of reads: 26507
Number of pairs: 4542239
Density: 0.01293
Diameter: NA
Mean edge weigth: 116.62
Max. degree: 1312

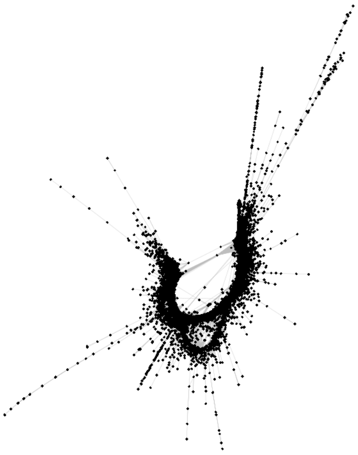

**CL21**

Number of reads: 25932
Number of pairs: 11941912
Density: 0.03552
Diameter: NA
Mean edge weigth: 119.49
Max. degree: 2134

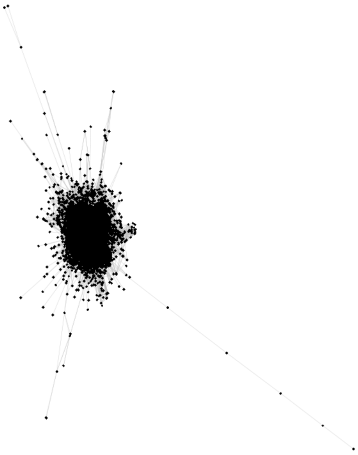

**CL22**

Number of reads: 25856
Number of pairs: 21767062
Density: 0.06512
Diameter: NA
Mean edge weigth: 123.91
Max. degree: 4587

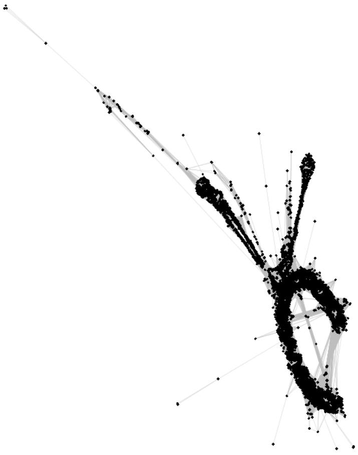

**CL23**

Number of reads: 21763
Number of pairs: 24985346
Density: 0.1055
Diameter: NA
Mean edge weigth: 129.75
Max. degree: 3658

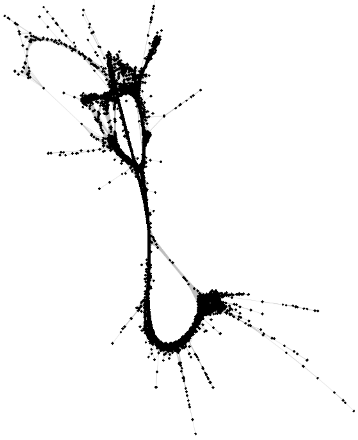

**CL24**

Number of reads: 25041
Number of pairs: 6852628
Density: 0.02186
Diameter: NA
Mean edge weigth: 125.1
Max. degree: 2330

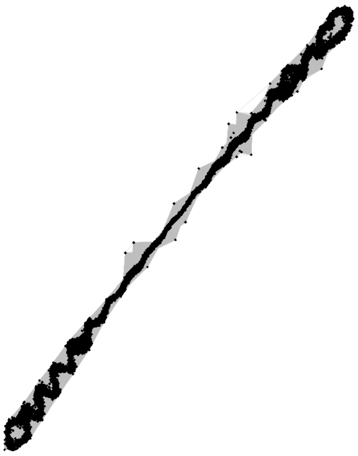

**CL25**

Number of reads: 20053
Number of pairs: 24977636
Density: 0.1242
Diameter: NA
Mean edge weigth: 154.71
Max. degree: 2835

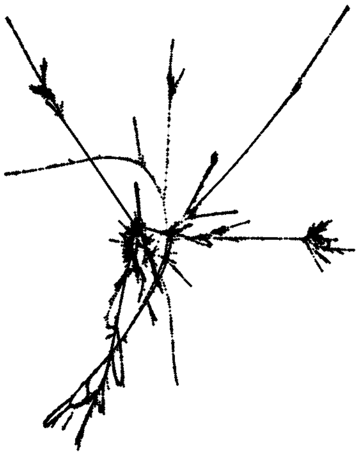

**CL26**

Number of reads: 23843
Number of pairs: 909631
Density: 0.0032
Diameter: NA
Mean edge weigth: 135.49
Max. degree: 463

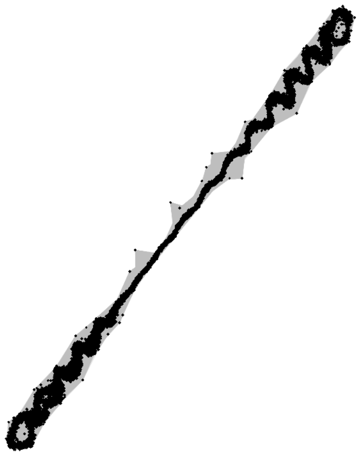

**CL27**

Number of reads: 19363
Number of pairs: 25026324
Density: 0.1335
Diameter: NA
Mean edge weigth: 154.59
Max. degree: 3012

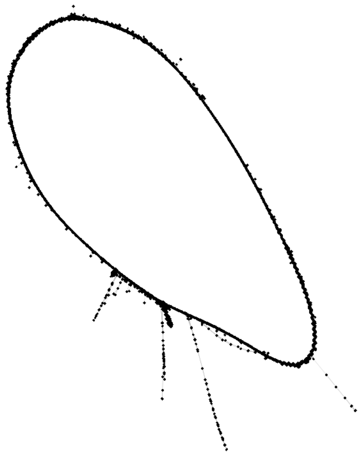

**CL28**

Number of reads: 23748
Number of pairs: 4003990
Density: 0.0142
Diameter: NA
Mean edge weigth: 151.75
Max. degree: 613

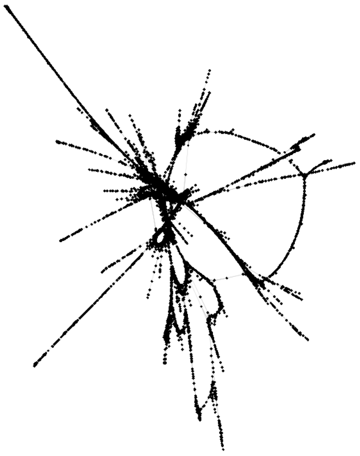

**CL29**

Number of reads: 23385
Number of pairs: 2348269
Density: 0.008589
Diameter: NA
Mean edge weigth: 122.82
Max. degree: 696

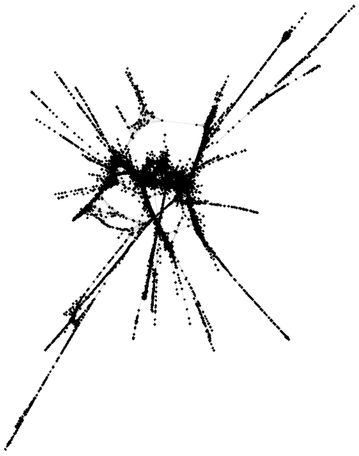

**CL30**

Number of reads: 23215
Number of pairs: 1547039
Density: 0.005741
Diameter: NA
Mean edge weigth: 111.44
Max. degree: 754

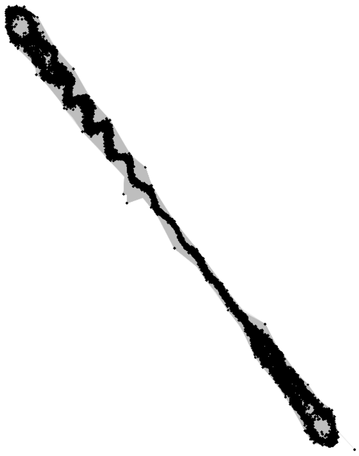

**CL31**

Number of reads: 18362
Number of pairs: 24956416
Density: 0.148
Diameter: NA
Mean edge weigth: 154.18
Max. degree: 3130

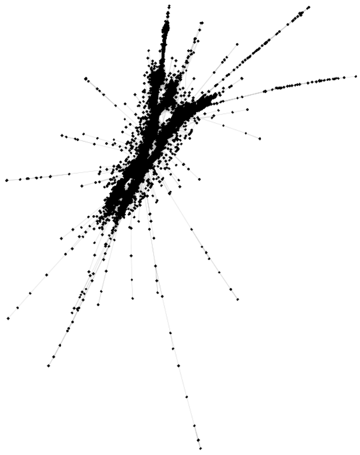

**CL32**

Number of reads: 23057
Number of pairs: 10526129
Density: 0.0396
Diameter: NA
Mean edge weigth: 129.51
Max. degree: 2357

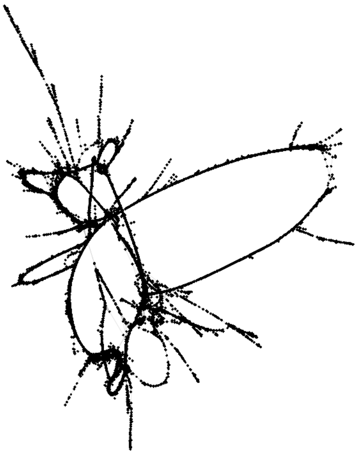

**CL33**

Number of reads: 22397
Number of pairs: 930694
Density: 0.003711
Diameter: NA
Mean edge weigth: 138.93
Max. degree: 340

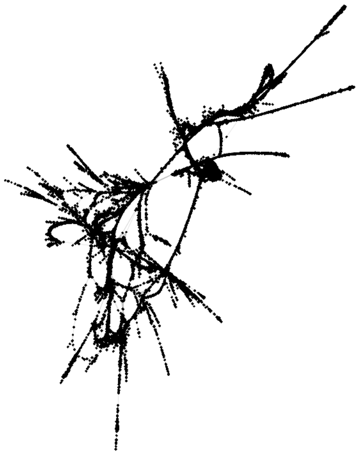

**CL34**

Number of reads: 22187
Number of pairs: 650652
Density: 0.002644
Diameter: NA
Mean edge weigth: 128.66
Max. degree: 241

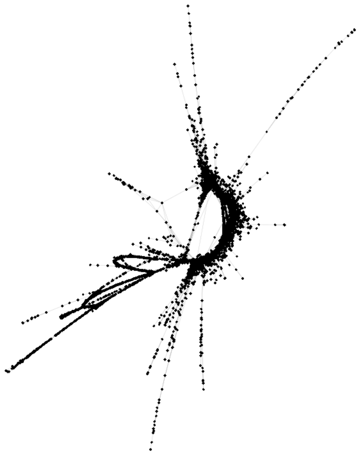

**CL35**

Number of reads: 21176
Number of pairs: 7047081
Density: 0.03143
Diameter: NA
Mean edge weigth: 117.44
Max. degree: 1700

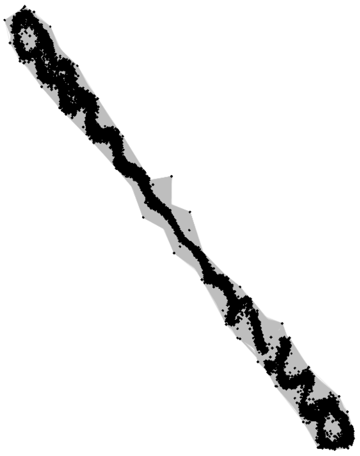

**CL36**

Number of reads: 17057
Number of pairs: 25010146
Density: 0.1719
Diameter: NA
Mean edge weigth: 155.14
Max. degree: 3332

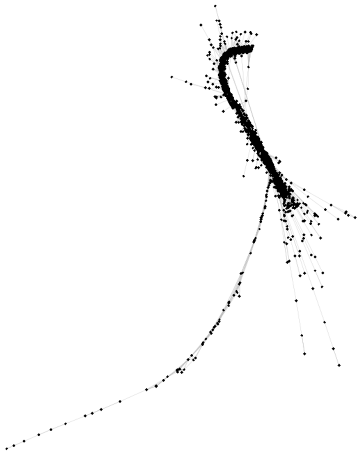

**CL37**

Number of reads: 20574
Number of pairs: 18469459
Density: 0.08727
Diameter: NA
Mean edge weigth: 143.3
Max. degree: 2291

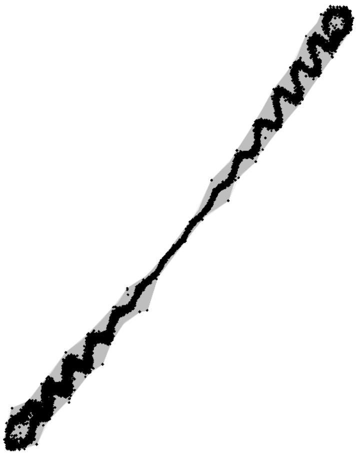

**CL38**

Number of reads: 18634
Number of pairs: 25007179
Density: 0.144
Diameter: NA
Mean edge weigth: 154.76
Max. degree: 3061

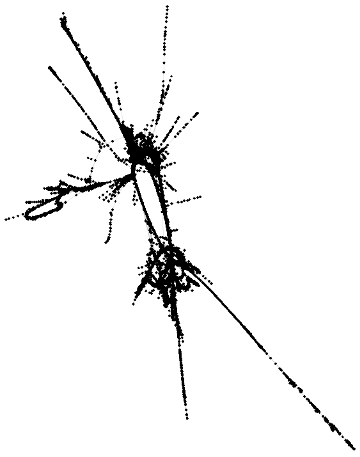

**CL39**

Number of reads: 20237
Number of pairs: 1480003
Density: 0.007228
Diameter: NA
Mean edge weigth: 125.63
Max. degree: 557

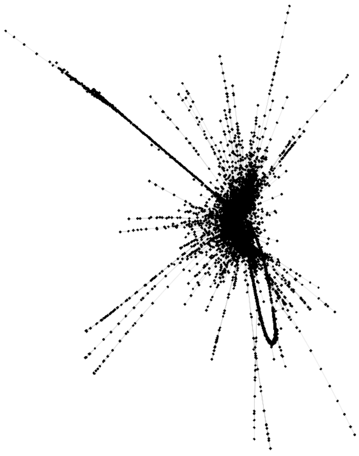

**CL40**

Number of reads: 20227
Number of pairs: 6156618
Density: 0.0301
Diameter: NA
Mean edge weigth: 121.41
Max. degree: 2489

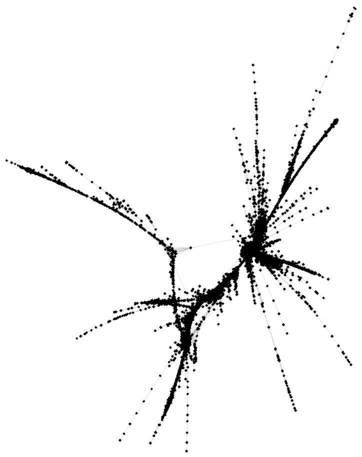

**CL41**

Number of reads: 20192
Number of pairs: 4261449
Density: 0.0209
Diameter: NA
Mean edge weigth: 121.43
Max. degree: 1709

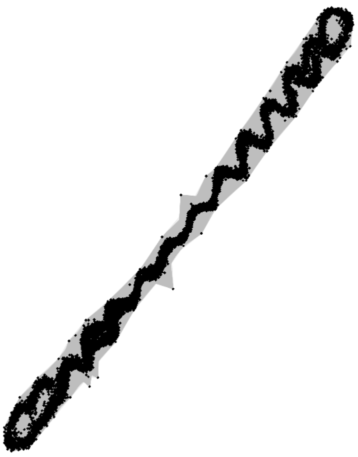

**CL42**

Number of reads: 17435
Number of pairs: 25004487
Density: 0.1645
Diameter: NA
Mean edge weigth: 154.76
Max. degree: 3206

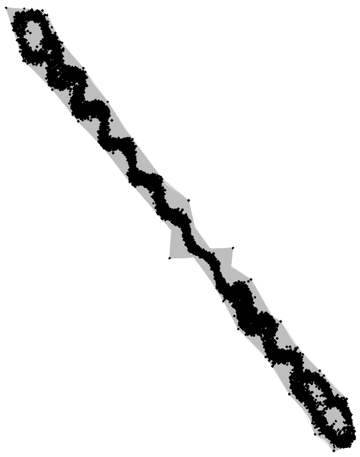

**CL43**

Number of reads: 16915
Number of pairs: 25003172
Density: 0.1748
Diameter: NA
Mean edge weigth: 154.93
Max. degree: 3317

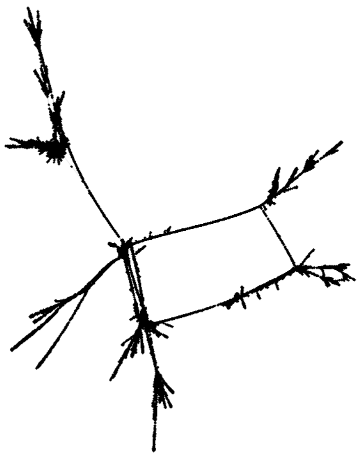

**CL44**

Number of reads: 19790
Number of pairs: 364658
Density: 0.001862
Diameter: NA
Mean edge weigth: 136.93
Max. degree: 245

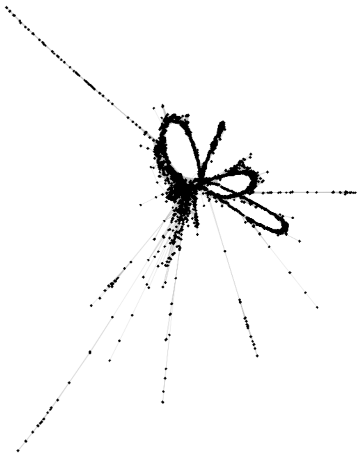

**CL45**

Number of reads: 19670
Number of pairs: 7461731
Density: 0.03857
Diameter: NA
Mean edge weigth: 141.4
Max. degree: 2278

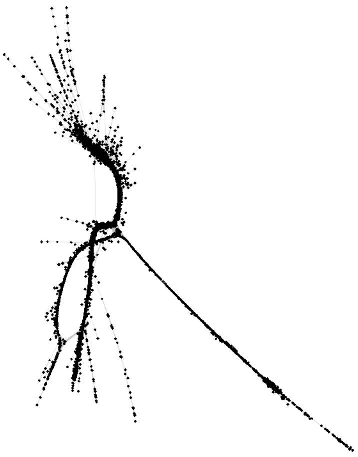

**CL46**

Number of reads: 19670
Number of pairs: 6322189
Density: 0.03268
Diameter: NA
Mean edge weigth: 133.95
Max. degree: 1962

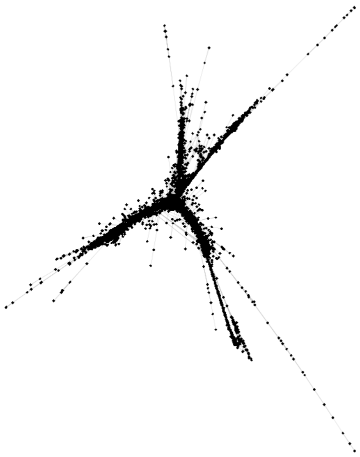

**CL47**

Number of reads: 19619
Number of pairs: 8695928
Density: 0.04519
Diameter: NA
Mean edge weigth: 122.93
Max. degree: 1907

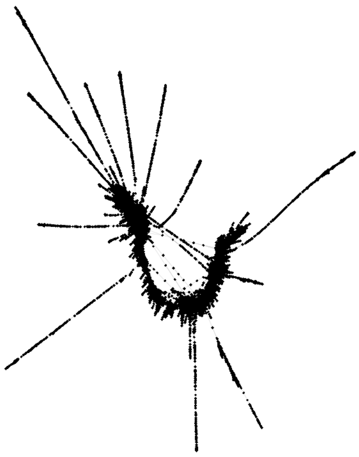

**CL48**

Number of reads: 19581
Number of pairs: 96522
Density: 0.0005035
Diameter: NA
Mean edge weigth: 104.11
Max. degree: 400

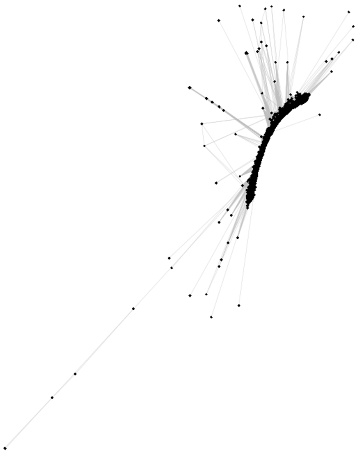

**CL49**

Number of reads: 16791
Number of pairs: 24999977
Density: 0.1774
Diameter: NA
Mean edge weigth: 148.69
Max. degree: 3520

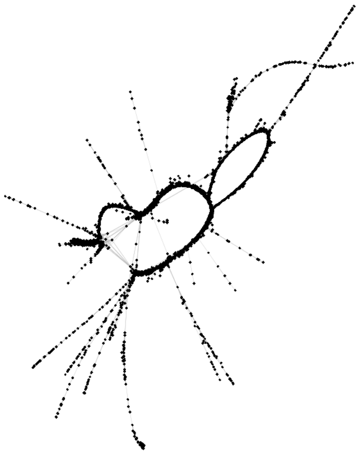

**CL50**

Number of reads: 18972
Number of pairs: 3598491
Density: 0.02
Diameter: NA
Mean edge weigth: 134.35
Max. degree: 812

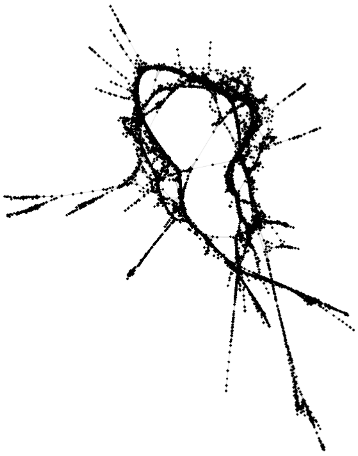

**CL51**

Number of reads: 18537
Number of pairs: 704072
Density: 0.004098
Diameter: NA
Mean edge weigth: 123.54
Max. degree: 259

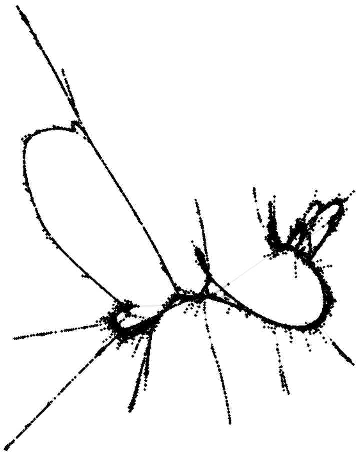

**CL52**

Number of reads: 18238
Number of pairs: 728825
Density: 0.004383
Diameter: NA
Mean edge weigth: 124.74
Max. degree: 288

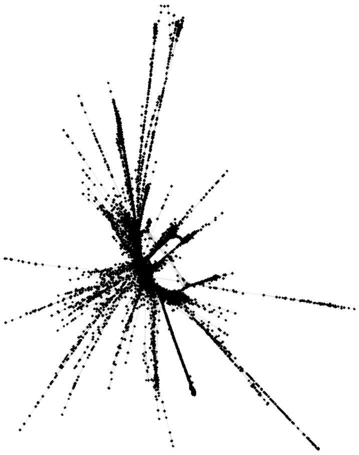

**CL53**

Number of reads: 18203
Number of pairs: 2714826
Density: 0.01639
Diameter: NA
Mean edge weigth: 128.25
Max. degree: 1629

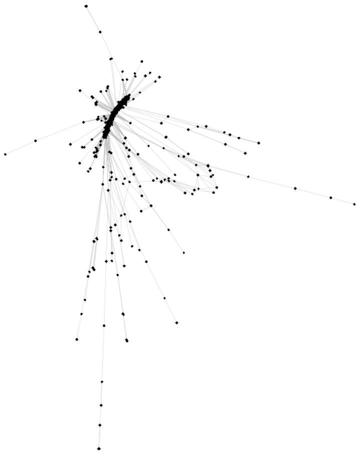

**CL54**

Number of reads: 16886
Number of pairs: 25014458
Density: 0.1755
Diameter: NA
Mean edge weigth: 149.04
Max. degree: 3487

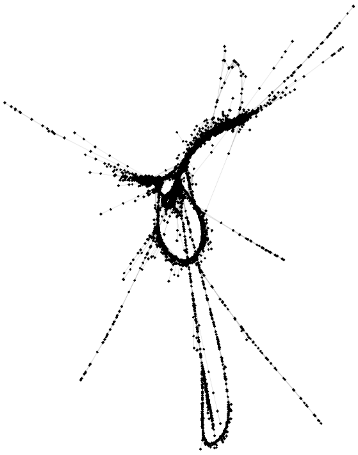

**CL55**

Number of reads: 18033
Number of pairs: 6454372
Density: 0.0397
Diameter: NA
Mean edge weigth: 128.4
Max. degree: 1551

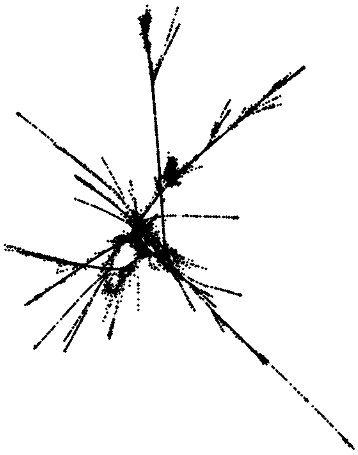

**CL56**

Number of reads: 17981
Number of pairs: 741079
Density: 0.004584
Diameter: NA
Mean edge weigth: 122.41
Max. degree: 487

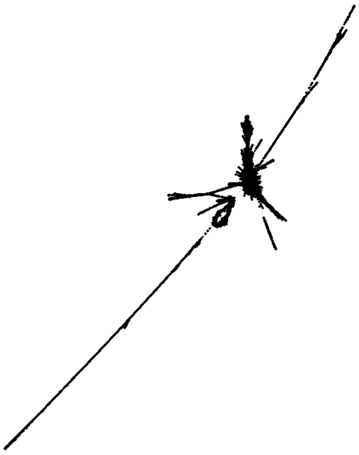

**CL57**

Number of reads: 17649
Number of pairs: 295203
Density: 0.001896
Diameter: NA
Mean edge weigth: 104.97
Max. degree: 1050

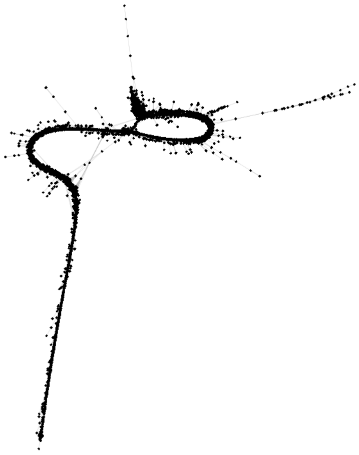

**CL58**

Number of reads: 17377
Number of pairs: 4395520
Density: 0.02911
Diameter: NA
Mean edge weigth: 138.12
Max. degree: 1139

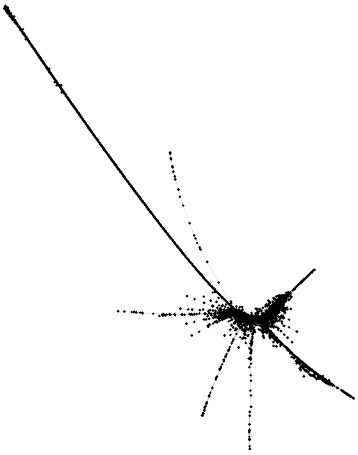

**CL59**

Number of reads: 17287
Number of pairs: 6351871
Density: 0.04251
Diameter: NA
Mean edge weigth: 127.76
Max. degree: 1935

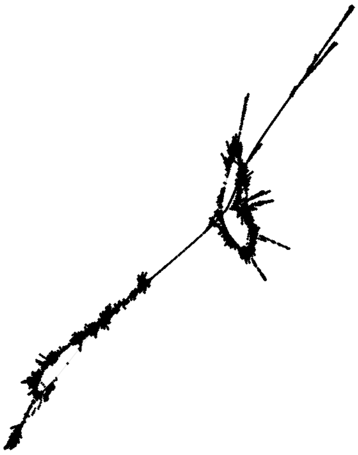

**CL60**

Number of reads: 17071
Number of pairs: 85850
Density: 0.0005892
Diameter: NA
Mean edge weigth: 116.86
Max. degree: 185

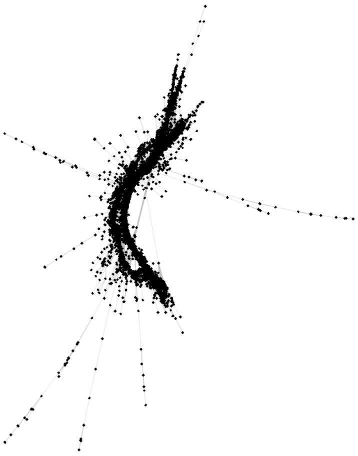

**CL61**

Number of reads: 16919
Number of pairs: 6411973
Density: 0.0448
Diameter: NA
Mean edge weigth: 127.48
Max. degree: 1586

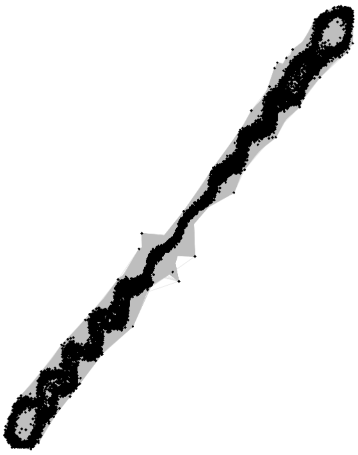

**CL62**

Number of reads: 16598
Number of pairs: 23959546
Density: 0.1739
Diameter: NA
Mean edge weigth: 154.81
Max. degree: 3334

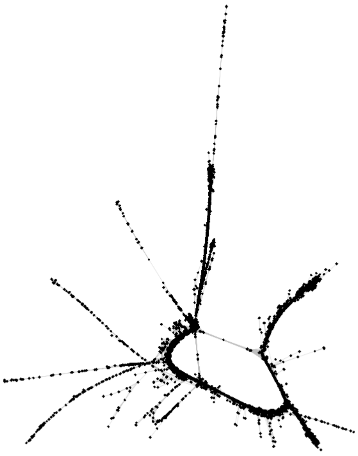

**CL63**

Number of reads: 16502
Number of pairs: 3930976
Density: 0.02887
Diameter: NA
Mean edge weigth: 144.42
Max. degree: 884

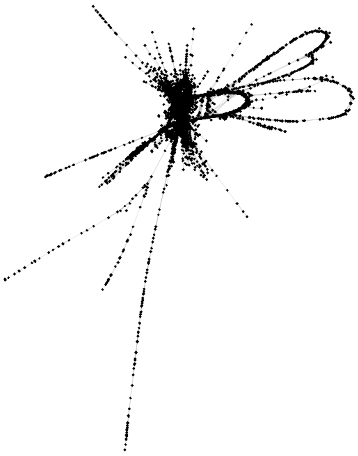

**CL64**

Number of reads: 16355
Number of pairs: 3108263
Density: 0.02324
Diameter: NA
Mean edge weigth: 116.98
Max. degree: 1719

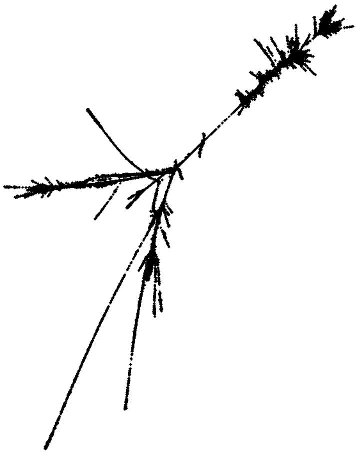

**CL65**

Number of reads: 16248
Number of pairs: 564837
Density: 0.004279
Diameter: NA
Mean edge weigth: 116.39
Max. degree: 407

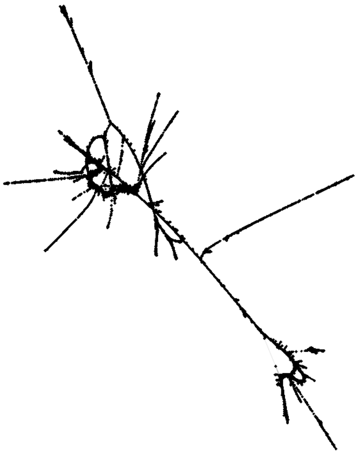

**CL66**

Number of reads: 16242
Number of pairs: 521391
Density: 0.003953
Diameter: NA
Mean edge weigth: 121.7
Max. degree: 265

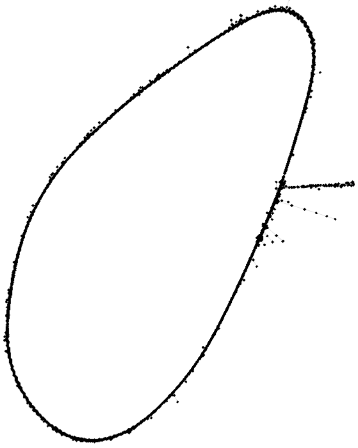

**CL67**

Number of reads: 15954
Number of pairs: 1632215
Density: 0.01283
Diameter: NA
Mean edge weigth: 153.1
Max. degree: 429

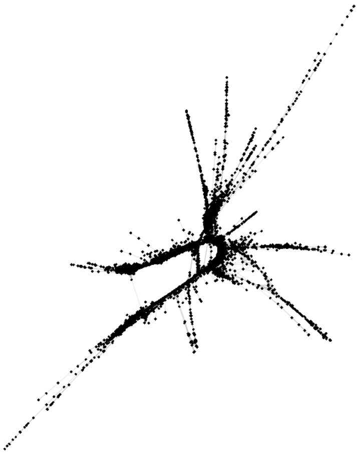

**CL68**

Number of reads: 15942
Number of pairs: 4425306
Density: 0.03483
Diameter: NA
Mean edge weigth: 133.83
Max. degree: 1041

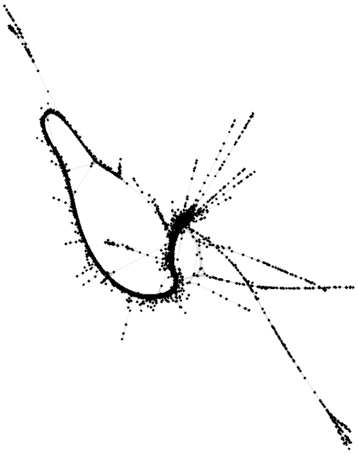

**CL69**

Number of reads: 15932
Number of pairs: 3090369
Density: 0.02435
Diameter: NA
Mean edge weigth: 134.78
Max. degree: 1268

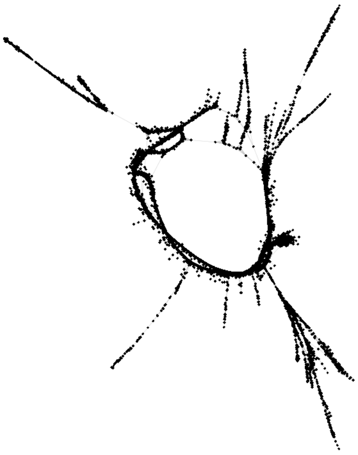

**CL70**

Number of reads: 15875
Number of pairs: 1470193
Density: 0.01167
Diameter: NA
Mean edge weigth: 138.6
Max. degree: 563

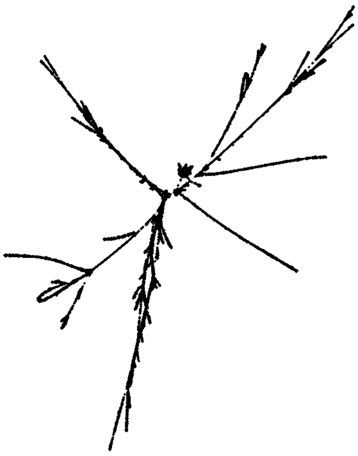

**CL71**

Number of reads: 15821
Number of pairs: 206986
Density: 0.001654
Diameter: NA
Mean edge weigth: 129.4
Max. degree: 205

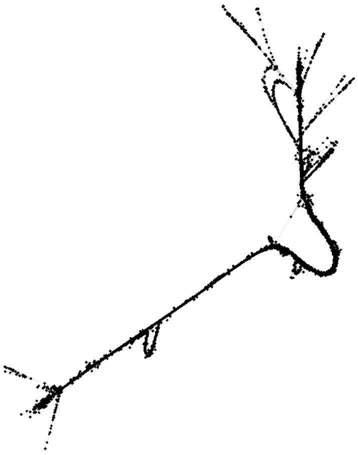

**CL72**

Number of reads: 15805
Number of pairs: 1944166
Density: 0.01557
Diameter: NA
Mean edge weigth: 139.75
Max. degree: 435

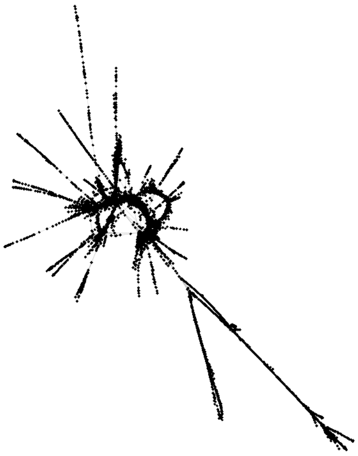

**CL73**

Number of reads: 15730
Number of pairs: 778580
Density: 0.006294
Diameter: NA
Mean edge weigth: 114.5
Max. degree: 630

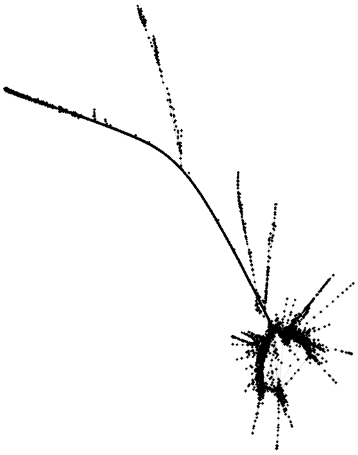

**CL74**

Number of reads: 15692
Number of pairs: 1693258
Density: 0.01375
Diameter: NA
Mean edge weigth: 128.72
Max. degree: 726

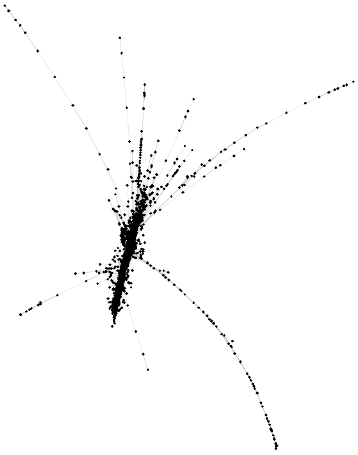

**CL75**

Number of reads: 15660
Number of pairs: 11439304
Density: 0.0933
Diameter: NA
Mean edge weigth: 124.24
Max. degree: 2819

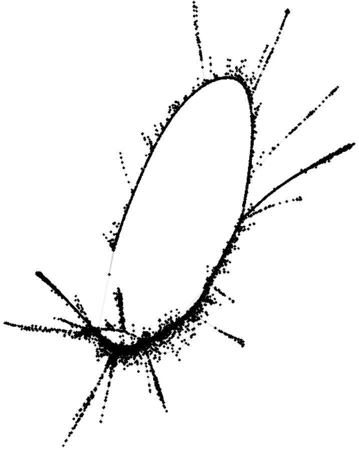

**CL76**

Number of reads: 15586
Number of pairs: 2049073
Density: 0.01687
Diameter: NA
Mean edge weigth: 136.66
Max. degree: 783

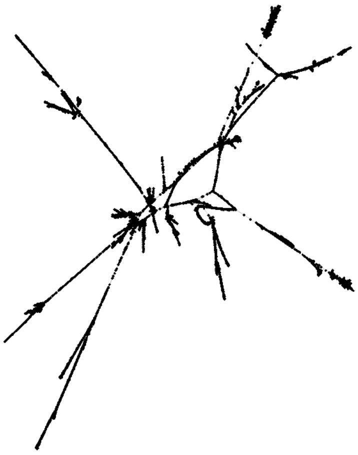

**CL77**

Number of reads: 15366
Number of pairs: 192334
Density: 0.001629
Diameter: NA
Mean edge weigth: 125.38
Max. degree: 187

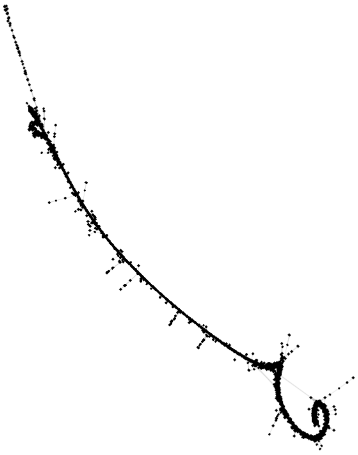

**CL78**

Number of reads: 15194
Number of pairs: 2584341
Density: 0.02239
Diameter: NA
Mean edge weigth: 142.55
Max. degree: 471

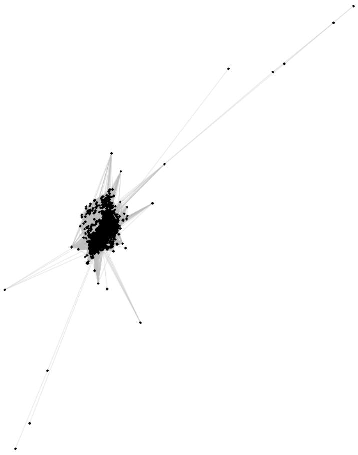

**CL79**

Number of reads: 10783
Number of pairs: 25061348
Density: 0.4311
Diameter: NA
Mean edge weigth: 135.04
Max. degree: 6222

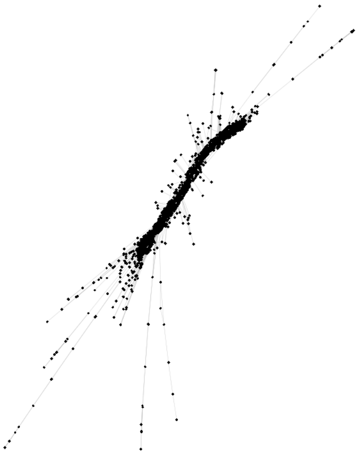

**CL80**

Number of reads: 14983
Number of pairs: 12439301
Density: 0.1108
Diameter: NA
Mean edge weigth: 143.49
Max. degree: 2092

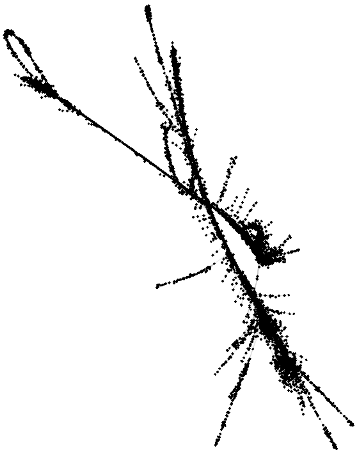

**CL81**

Number of reads: 14884
Number of pairs: 1724321
Density: 0.01557
Diameter: NA
Mean edge weigth: 132.47
Max. degree: 749

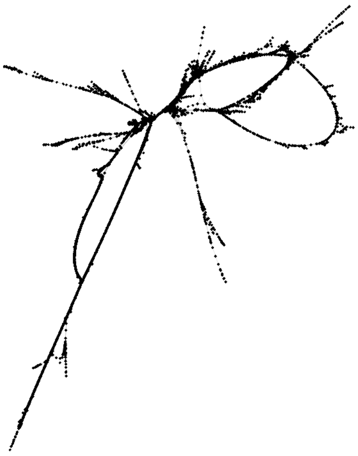

**CL82**

Number of reads: 14743
Number of pairs: 1707725
Density: 0.01571
Diameter: NA
Mean edge weigth: 142.85
Max. degree: 1308

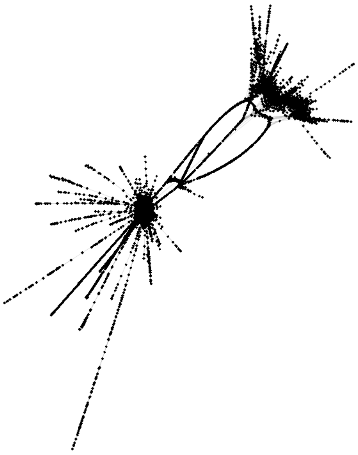

**CL83**

Number of reads: 14471
Number of pairs: 2969693
Density: 0.02836
Diameter: NA
Mean edge weigth: 104.92
Max. degree: 2642

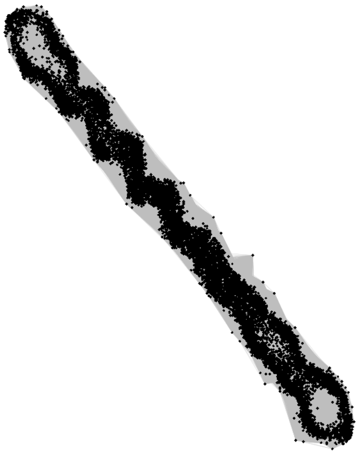

**CL84**

Number of reads: 14449
Number of pairs: 23711094
Density: 0.2272
Diameter: NA
Mean edge weigth: 154.92
Max. degree: 3712

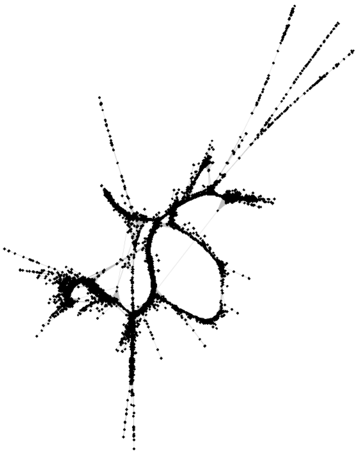

**CL85**

Number of reads: 13861
Number of pairs: 1809445
Density: 0.01884
Diameter: NA
Mean edge weigth: 123.59
Max. degree: 688

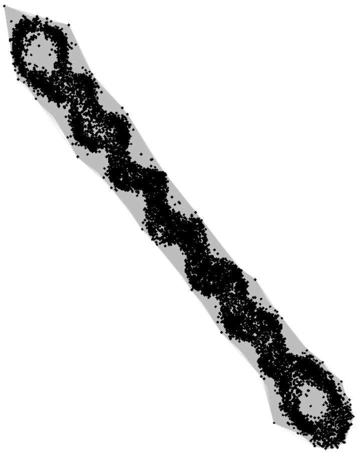

**CL86**

Number of reads: 13524
Number of pairs: 21986762
Density: 0.2404
Diameter: NA
Mean edge weigth: 155.37
Max. degree: 3708

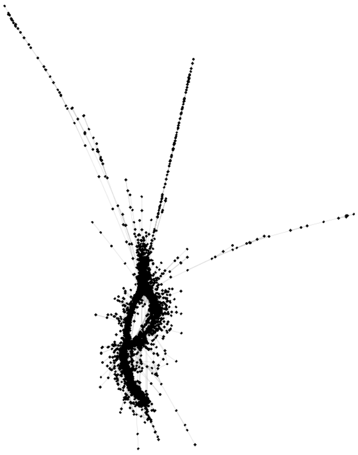

**CL87**

Number of reads: 13153
Number of pairs: 4373358
Density: 0.05056
Diameter: NA
Mean edge weigth: 121.32
Max. degree: 1601

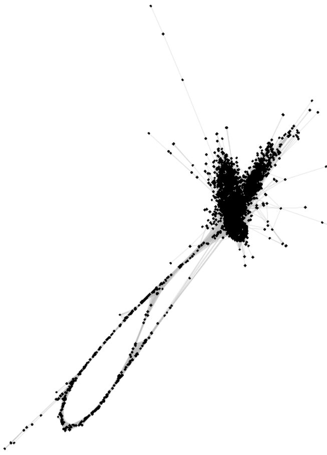

**CL88**

Number of reads: 13050
Number of pairs: 14598552
Density: 0.1715
Diameter: NA
Mean edge weigth: 121.84
Max. degree: 8962

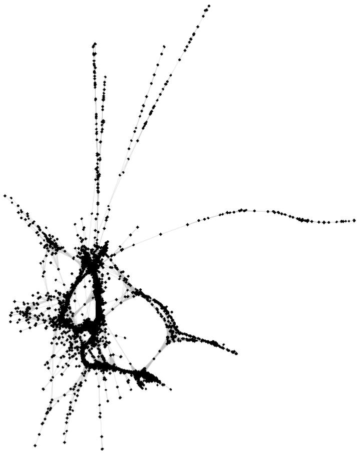

**CL89**

Number of reads: 12112
Number of pairs: 1788939
Density: 0.02439
Diameter: NA
Mean edge weigth: 129.59
Max. degree: 1301

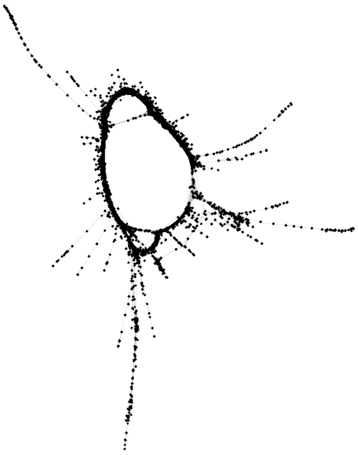

**CL90**

Number of reads: 12041
Number of pairs: 1444927
Density: 0.01993
Diameter: NA
Mean edge weigth: 121.91
Max. degree: 562

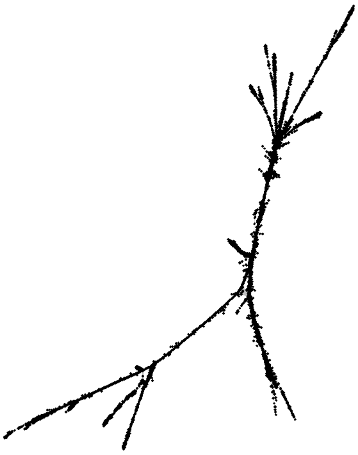

**CL91**

Number of reads: 11746
Number of pairs: 704494
Density: 0.01021
Diameter: NA
Mean edge weigth: 132.7
Max. degree: 325

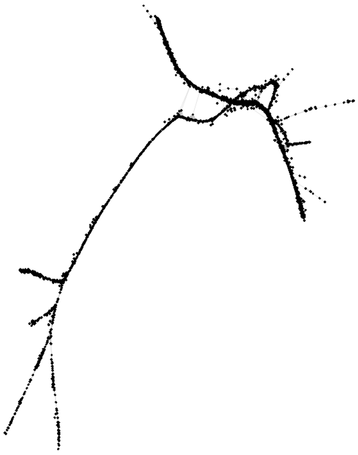

**CL92**

Number of reads: 11591
Number of pairs: 1553625
Density: 0.02313
Diameter: NA
Mean edge weigth: 143.46
Max. degree: 433

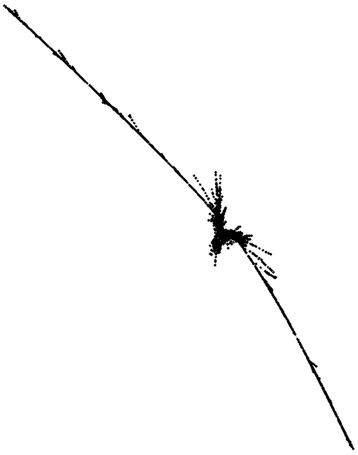

**CL93**

Number of reads: 11565
Number of pairs: 1763131
Density: 0.02637
Diameter: NA
Mean edge weigth: 112.34
Max. degree: 959

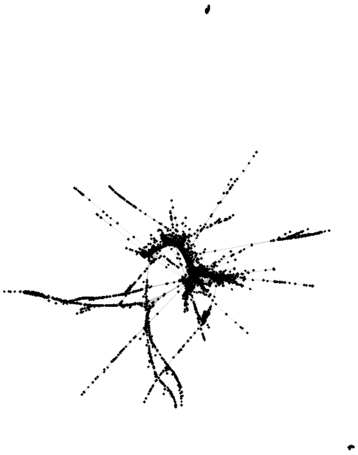

**CL94**

Number of reads: 11500
Number of pairs: 872418
Density: 0.01319
Diameter: NA
Mean edge weigth: 114.83
Max. degree: 634

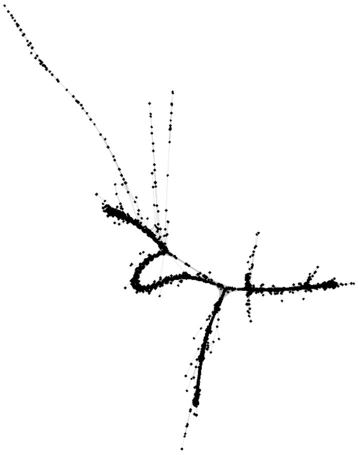

**CL95**

Number of reads: 11387
Number of pairs: 2414715
Density: 0.03725
Diameter: NA
Mean edge weigth: 145.62
Max. degree: 882

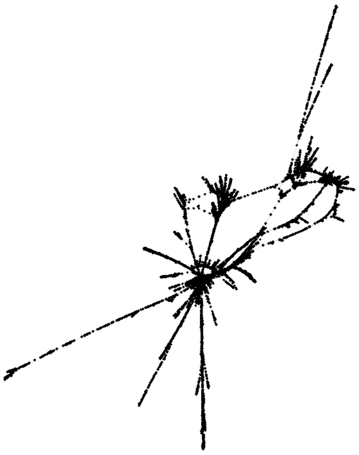

**CL96**

Number of reads: 11331
Number of pairs: 339980
Density: 0.005296
Diameter: NA
Mean edge weigth: 139.67
Max. degree: 355

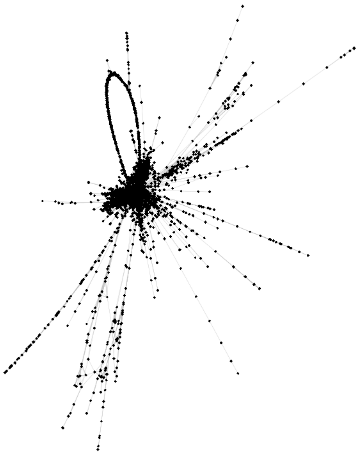

**CL97**

Number of reads: 11003
Number of pairs: 4644549
Density: 0.07673
Diameter: NA
Mean edge weigth: 128.55
Max. degree: 2475

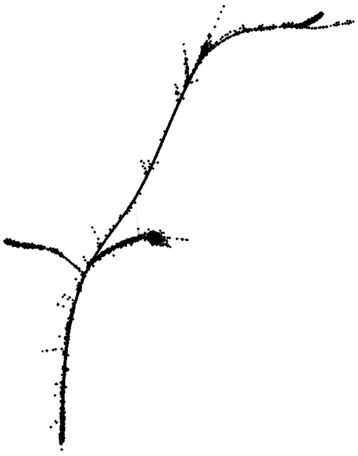

**CL98**

Number of reads: 10966
Number of pairs: 1434705
Density: 0.02386
Diameter: NA
Mean edge weigth: 148.59
Max. degree: 453

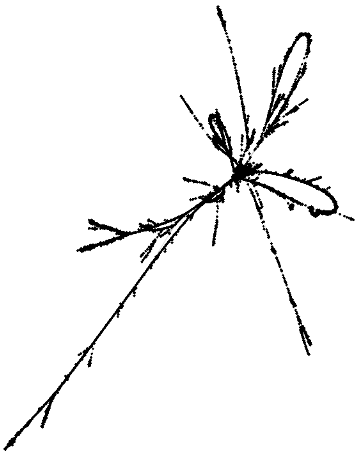

**CL99**

Number of reads: 10928
Number of pairs: 264198
Density: 0.004425
Diameter: NA
Mean edge weigth: 133.01
Max. degree: 206

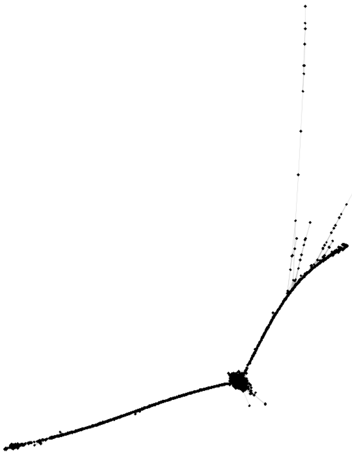

**CL100**

Number of reads: 10897
Number of pairs: 11774251
Density: 0.1983
Diameter: NA
Mean edge weigth: 120.34
Max. degree: 5535

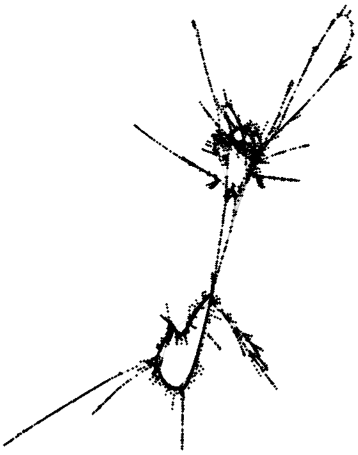

**CL101**

Number of reads: 10853
Number of pairs: 376423
Density: 0.006392
Diameter: NA
Mean edge weigth: 134.28
Max. degree: 223

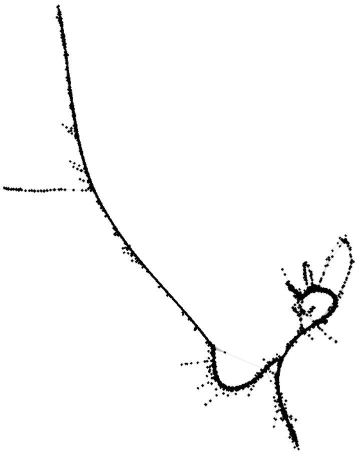

**CL102**

Number of reads: 10823
Number of pairs: 1126365
Density: 0.01923
Diameter: NA
Mean edge weigth: 146.53
Max. degree: 454

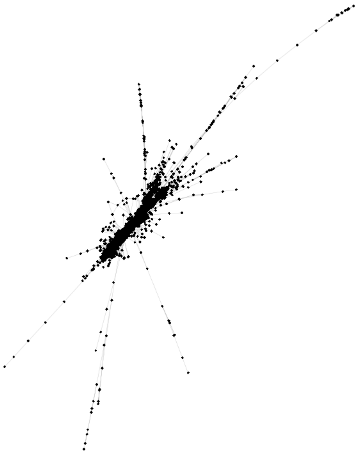

**CL103**

Number of reads: 10788
Number of pairs: 6338727
Density: 0.1089
Diameter: NA
Mean edge weigth: 120.7
Max. degree: 2106

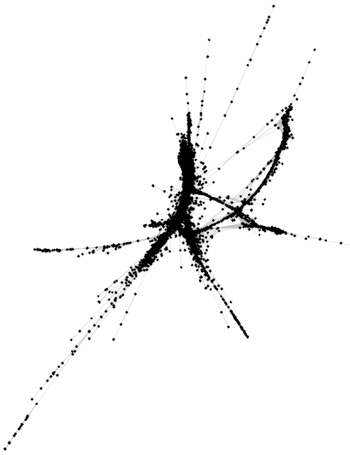

**CL104**

Number of reads: 10677
Number of pairs: 2397533
Density: 0.04207
Diameter: NA
Mean edge weigth: 118.71
Max. degree: 1654

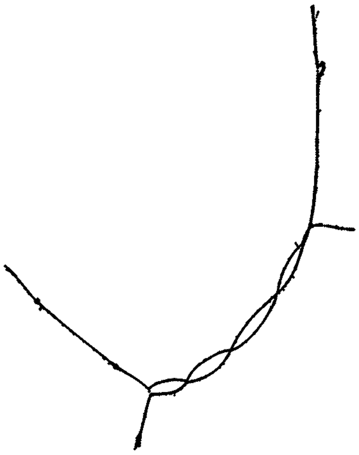

**CL105**

Number of reads: 10589
Number of pairs: 253094
Density: 0.004515
Diameter: NA
Mean edge weigth: 138.49
Max. degree: 107

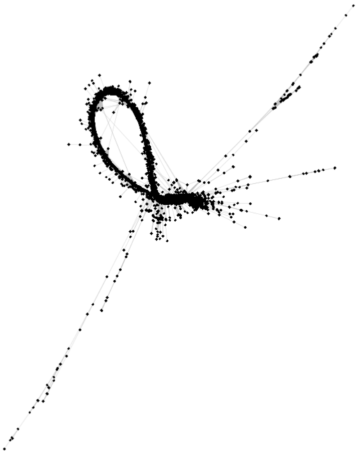

**CL106**

Number of reads: 10535
Number of pairs: 3471658
Density: 0.06257
Diameter: NA
Mean edge weigth: 128.68
Max. degree: 1455

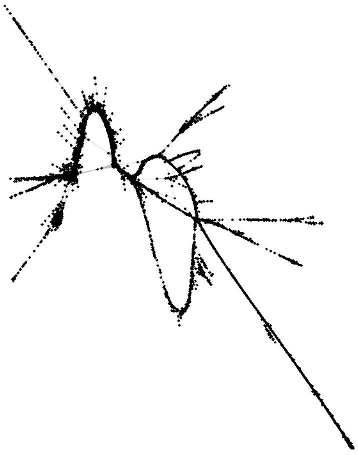

**CL107**

Number of reads: 10451
Number of pairs: 568732
Density: 0.01042
Diameter: NA
Mean edge weigth: 124.94
Max. degree: 412

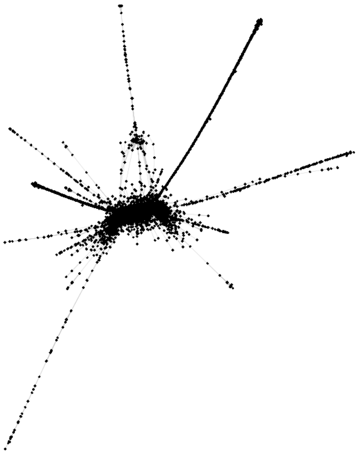

**CL108**

Number of reads: 10366
Number of pairs: 1515436
Density: 0.02821
Diameter: NA
Mean edge weigth: 114.72
Max. degree: 1370

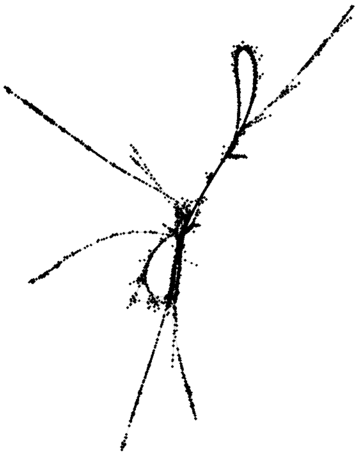

**CL109**

Number of reads: 10342
Number of pairs: 1316587
Density: 0.02462
Diameter: NA
Mean edge weigth: 135.46
Max. degree: 628

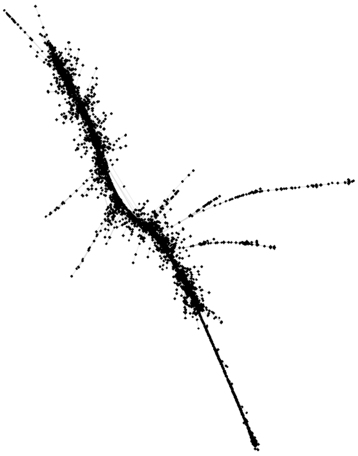

**CL110**

Number of reads: 10289
Number of pairs: 1484013
Density: 0.02804
Diameter: NA
Mean edge weigth: 134.05
Max. degree: 637

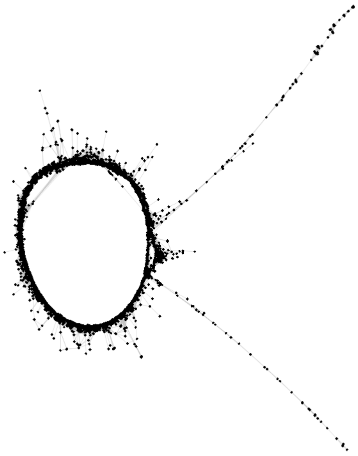

**CL111**

Number of reads: 10261
Number of pairs: 1664297
Density: 0.03162
Diameter: NA
Mean edge weigth: 129.77
Max. degree: 533

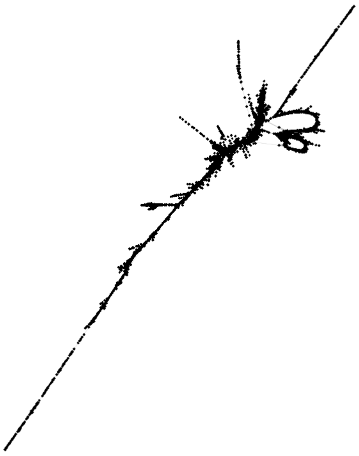

**CL112**

Number of reads: 10186
Number of pairs: 450315
Density: 0.008681
Diameter: NA
Mean edge weigth: 117.55
Max. degree: 394

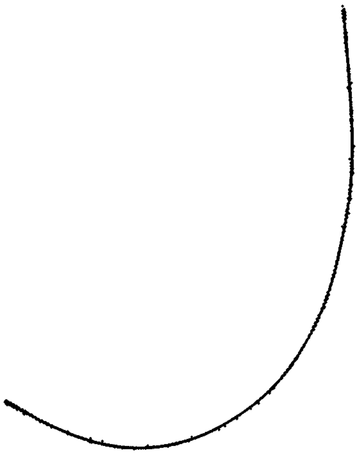

**CL113**

Number of reads: 10122
Number of pairs: 771262
Density: 0.01506
Diameter: NA
Mean edge weigth: 154.21
Max. degree: 217

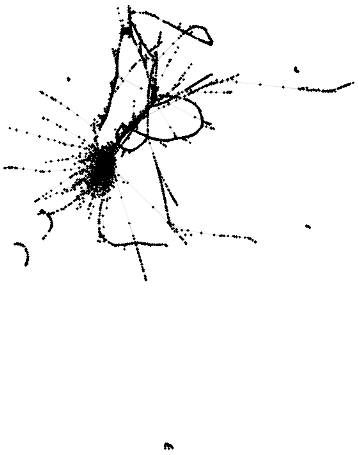

**CL114**

Number of reads: 10031
Number of pairs: 537065
Density: 0.01068
Diameter: NA
Mean edge weigth: 115.59
Max. degree: 732
