# Supplementary material for: Landscape of Transposable Elements Focusing on the B Chromosome of the Cichlid Fish Astatotilapia latifasciata
Source: Genes (Basel). 2018 May 23;9(6):269. doi: 10.3390/genes9060269 (PMC6027319; doi:10.3390/genes9060269)
Supplement: Supplementary file 1 [file genes-09-00269-s001.zip › SupplementaryFigure1.docx]

**Supplementary Figure 1.** Repetitive element transcription levels quantified by RPKM metrics for brain, muscle and male and female gonads of B- individuals. The graph shows repeat superfamiles which includes various families with their respective copies. Each boxplot represents a range of expression values (log2 RPKM) from all expressed copies in the tissue. Although some superfamiles have only one highly expressed copy (srpRNA) most shows wide transcription distribution. The distinct superfamilies analyzed are indicated in the bottom.

**
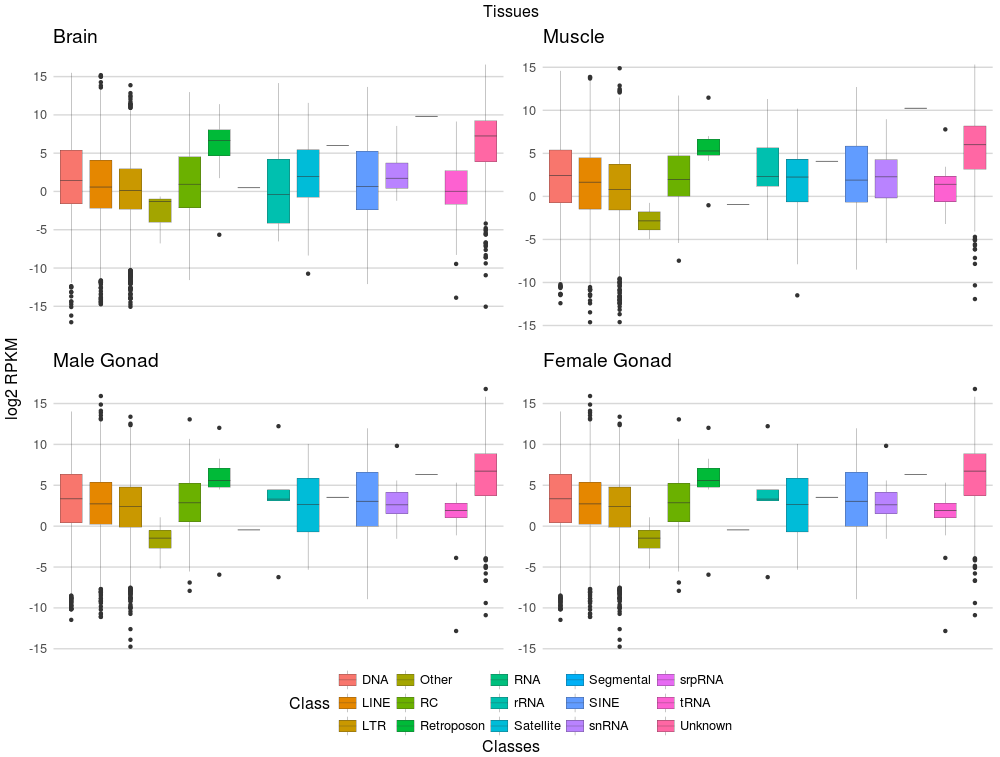
**
