# Supplementary material for: Landscape of Transposable Elements Focusing on the B Chromosome of the Cichlid Fish Astatotilapia latifasciata
Source: Genes (Basel). 2018 May 23;9(6):269. doi: 10.3390/genes9060269 (PMC6027319; doi:10.3390/genes9060269)
Supplement: Supplementary file 1 [file genes-09-00269-s001.zip › SupplementaryMethods.docx]

­Article

Landscape of Transposable Elements Focusing on the B Chromosome of the Cichlid Fish *Astatotilapia latifasciata*

Rafael L. B. Coan and Cesar Martins *

**Supplementary Methods**

Determination of cut-off for coverage regions

The single copy gene hypoxanthine phosphoribosyltransferase (HPRT) was used as reference to calculate coverage ratios and qPCR normalization. Coverage from the different genomes were always normalized considering the coverage ratio of the HPRT region on those genomes.

To find the HPRT gene in our recently assembled genome we extracted its sequence from *Metriaclima zebra* available in Sacibase (<http://sacibase.ibb.unesp.br>). With this sequence we performed a BLAST (Altschul et al. 1990) on *A. latifasciata* assembled genome. Region 11,026 to 15,706 from “NODE_61732” returned high similarity. To confirm that the region belongs to HPRT a recently assembled transcriptome of the species (Marques, 2016) with gmap 2016-06-09 (Wu & Watanabe 2005) was explored. A functional annotation of the mapped transcript with Trinotate 2.0.2 (Haas et al. 2013) was used to confirm the gene annotation. After extraction of per base coverage from the HPRT region we used R to calculate mean, standard deviation and median for all samples (Table S2). For each alignment we used the mean coverage value plus standard deviation as cut-off for transposable elements coverage calculation. Only regions with coverage higher than HPRT gene coverage were considered. Only repeat regions with higher coverage than a typical gene were used in the analysis.

We then calculated ratios between HPRT coverage for all alignments using the reads dataset M1-0B as reference (Table S3). We chose this sample as reference due to its good sequencing quality and previous analysis by our group (Valente et al. 2014). M1-0B coverage values were used to normalize the differences sequencing coverages thus creating a common ground for comparing the various samples.

Proportions in Table S3 shows two levels of normalization. First, they are normalized by the sequencing coverage; second, we calculated the ratios between the given sample and the M1-0B reference. In this way table S3 shows the coverage ratio between a given TE. Therefore we can calculate how many more copies an element has in comparison to the M1-0B reference. This approach can indicates repetitive elements with higher number of copies in the B chromosome.

References

1. Altschul S.F.; Gish W.; Miller W.; Myers E.W.; Lipman D.J. Basic local alignment search tool. J. Mol. Biol. 1990, 215, 403–10. doi: 10.1016/S0022-2836(05)80360-2.
2. Haas BJ et al. 2013. De novo transcript sequence reconstruction from RNA-seq using the Trinity platform for reference generation and analysis. Nat. Protoc. 2013, 8, 1494–512. doi: 10.1038/nprot.2013.084.
3. Marques, D.F. Functional analysis of B chromosome presence using cichlid Astatotilapia latifasciata as model. Available online: http://hdl.handle.net/11449/141953 (accessed on 12 March 2018).
4. 10. Valente, G.T.; Conte, M.A.; Fantinatti, B.E.A.; Cabral-de-Mello, D.C.; Carvalho, R.F.; Vicari, M.R.; Kocher, T.D.; Martins, C. Origin and evolution of B chromosomes in the cichlid fish Astatotilapia latifasciata based on integrated genomic analyses. Mol. Biol. Evol. 2014, 31, 2061–2072, doi:10.1093/molbev/msu148.
5. Wu T.D.; Watanabe C.K. GMAP: A genomic mapping and alignment program for mRNA and EST sequences. Bioinformatics 2005, 21, 1859–1875. doi: 10.1093/bioinformatics/bti310.
