# Supplementary material for: Landscape of Transposable Elements Focusing on the B Chromosome of the Cichlid Fish Astatotilapia latifasciata
Source: Genes (Basel). 2018 May 23;9(6):269. doi: 10.3390/genes9060269 (PMC6027319; doi:10.3390/genes9060269)
Supplement: Supplementary file 1 [file genes-09-00269-s001.zip › SupplementaryTablesS1-S7.docx]

­Article

Landscape of Transposable Elements Focusing on the B Chromosome of the Cichlid Fish *Astatotilapia latifasciata*

Rafael L. B. Coan and Cesar Martins *

**Supplementary Tables**

**Table S1**. Summary of most important information about the investigated samples. M, male; F, female; 0B, absence of B chromosomes; 1B, presence of one B chromosome; 2B, presence of two B chromosomes. For RNA sequencing (RNA-seq) we have analyzed 3 samples for each sex and B status.

| **Type of analysis** | **Tissue** | **B status (sample ID)** |
| --- | --- | --- |
| DNA-Seq | Liver | B- (M1-0B, F1-0B) |
|  | Liver | B+ (M2-1B, M3-1B, M4-2B, F2-1B) |
|  |  |  |
| RNA-Seq | Brain | B- (3M, 3F) |
|  | Brain | B+ (3M, 3F) |
|  | Muscle | B- (3M, 3F) |
|  | Muscle | B+ (3M, 3F) |
|  | Gonads | B- (3M, 3F) |
|  | Gonads | B+ (3M, 3F) |
|  |  |  |
| FISH | Kidney | B+ |
|  |  |  |
| qPCR | Caudal Fin | B- (04) |
|  | Caudal Fin | B+ (907, 908, 910, 913, 918, 919) |
|  |  |  |
| RepeatExplorer | Liver | B- (M1-0B) |
|  | Liver | B+ (M4-2B) |
|  |  |  |
| Coverage Ratios | Liver | B- (M1-0B, F1-0B) |
|  | Liver | B+ (M2-1B, M3-1B, M4-2B, F2-1B) |

**Table S2.** Samples and statistics for the six read dataset alignments against *A. latifasciata* reference genome.

| Sample | Total reads | Aligned reads | Read size |
| --- | --- | --- | --- |
| M1-0B | 353,134,494 | 342,255,717 | 101 |
| F1-0B | 221,854,697 | 208,523,209 | 191 |
| F2-1B | 209,344,332 | 194,934,049 | 191 |
| M2-1B | 123,397,225 | 119,723,749 | 101 |
| M3-1B | 306,683,003 | 297,361,258 | 101 |
| M4-2B | 272,496,627 | 261,694,628 | 101 |

**Table S3.** Metrics of the HPRT gene coverage for the six alignments.

| Sample | Mean coverage | Standard deviation | Median coverage |
| --- | --- | --- | --- |
| M1-0B | 39.14 | 10.15 | 39.0 |
| F1-0B | 54.42 | 11.76 | 53.0 |
| F2-1B | 49.60 | 12.06 | 51.0 |
| M2-1B | 15.20 | 5.05 | 15.0 |
| M3-1B | 35.38 | 9.0 | 35.0 |
| M4-2B | 29.92 | 9.37 | 29.0 |

**Table S4**. Ratios between HPRT coverages in reference to M1-0B dataset.

| Samples | Proportion | Standard deviation |
| --- | --- | --- |
| F1-0B/M1-0B | 1.47 | 0.47 |
| F2-0B/M1-0B | 1.32 | 0.42 |
| M2-1B/M1-0B | 0.40 | 0.13 |
| M3-1B/M1-0B | 0.94 | 0.25 |
| M4-2B/M1-0B | 0.78 | 0.20 |

**Table S5**. Primers used for the construction of custom FISH probes.

| **Element** | **Cluster** | **Forward (5'-3')** | **Reverse (5'-3')** | **Product (bp)** |
| --- | --- | --- | --- | --- |
| Bel/Pao | 28 | TATGACTCAGCCTGGAAGC | GTTGGGTGTCTCTTTTGGC | 659 |
| Gypsy | 67 | ATAAACTCCGTGTGATTGC | ATTGGTTGTGTGGTTCTTC | 477 |
| L2 | 74 | GGCAAGGACAGGGGTGATAG | TTGATGATTTGGTGGACTGG | 624 |
| DNA/hAT | 63 | GATGTATGCTGGGTAATGGC | GAGTGGATTCTGGAGTCTTC | 207 |

**Table S6**. Primers used for qPCR.

| **Element** | **Cluster** | **Forward (5'-3')** | **Reverse (5'-3')** | **Product (bp)** |
| --- | --- | --- | --- | --- |
| Bel/Pao | 28 | GCTTTGGATGTTTGAAGACC | GTTGGGTGTCTCTTTTGGC | 85 |
| Gypsy | 67 | ACTCCAGATTTCAACTCACC | ATTGGTTGTGTGGTTCTTC | 85 |

**Table S7.** Copy number estimates for several elements found in the *A.* *latifasciata* genome.

| **TEs** | **Copy Number** | **Bases Masked** | **Percentage** |
| --- | --- | --- | --- |
| LINEs |  |  |  |
| L1 | 21,187 | 5,144,393 | 0.68 |
| L2 | 92,444 | 18,489,818 | 2.44 |
| Penelope | 28,071 | 636 | 0.57 |
| RTE | 6,222 | 5,610 | 0.12 |
| RTE-BovB | 27,508 | 268 | 0.85 |
| Rex-Babar | 40,220 | 4,313,709 | 1.06 |
| Other | 19,525 | 19,434,447 | 0.54 |
| Total LINE | 235,177 | 47,388,881 | 6.26 |
|  | | | |
| LTRs |  |  |  |
| Copia | 1,286 | 253,519 | 0.03 |
| DIRS | 1,513 | 355,559 | 0.05 |
| ERV1 | 7,922 | 1,300,811 | 0.17 |
| Gypsy | 17,806 | 4,629,212 | 0.61 |
| Pao | 5,840 | 1,599,144 | 0.21 |
| Other | 18,097 | 1,614,864 | 0.21 |
| Total LTR | 52,464 | 9,753,109 | 1.28 |
|  |  |  |  |
| SINE | 66,647 | 8,140,121 | 1.06 |
|  | | | |
| DNA |  |  |  |
| PIF-Harbinger | 11,423 | 2,175,244 | 0.29 |
| PiggyBac | 12,866 | 2,729,557 | 0.36 |
| TcMar-Tc1 | 149,220 | 33,134,074 | 4.37 |
| hAT-Ac | 74,006 | 13,418,194 | 1.77 |
| hAT-Charlie | 37,310 | 6,901,211 | 0.91 |
| Other | 179,218 | 25,181,105 | 3,31 |
| Total DNA | 464,043 | 83,539,385 | 11.01 |
|  |  |  |  |
| Other Elements | 275,935 | 13,129,782 | 1.73 |
| Unclassified Elements | 322,699 | 53,635,854 | 7.07 |
|  |  |  |  |
| Total Genome | 1,416,965 | 215,587,132 | 28.41 |
